# Lessening the Impact of Financial Toxicity (LIFT): a protocol for a multi-site, single-arm trial examining the effect of financial navigation on financial toxicity in adult patients with cancer in rural and non-rural settings

**DOI:** 10.1186/s13063-022-06745-4

**Published:** 2022-10-03

**Authors:** Stephanie B. Wheeler, Caitlin B. Biddell, Michelle L. Manning, Mindy S. Gellin, Neda R. Padilla, Lisa P. Spees, Cynthia D. Rogers, Julia Rodriguez-O’Donnell, Cleo Samuel-Ryals, Sarah A. Birken, Katherine E. Reeder-Hayes, Victoria M. Petermann, Allison M. Deal, Donald L. Rosenstein

**Affiliations:** 1 Department of Health Policy and Management, University of North Carolina at Chapel Hill, Gillings School of Global Public Health, Chapel Hill, NC USA; 2 Lineberger Comprehensive Cancer Center, University of North Carolina at Chapel Hill, Chapel Hill, NC USA; 3 Wake Forest School of Medicine, Winston-Salem, NC USA; 4 Wake Forest Baptist Comprehensive Cancer Center, Winston-Salem, NC USA; 5 Division of Oncology, University of North Carolina at Chapel Hill, Chapel Hill, NC USA; 6 School of Nursing, University of North Carolina at Chapel Hill, Chapel Hill, NC USA; 7 Departments of Psychiatry and Medicine, University of North Carolina at Chapel Hill, Chapel Hill, NC USA

**Keywords:** Financial navigation, Financial toxicity, Multi-site behavioral intervention, Cancer

## Abstract

**Background:**

Almost half of the patients with cancer report cancer-related financial hardship, termed “financial toxicity” (FT), which affects health-related quality of life, care retention, and, in extreme cases, mortality. This increasingly prevalent hardship warrants urgent intervention. Financial navigation (FN) targets FT by systematically identifying patients at high risk, assessing eligibility for existing resources, clarifying treatment cost expectations, and working with patients and caregivers to develop a plan to cope with cancer costs. This trial seeks to (1) identify FN implementation determinants and implementation outcomes, and (2) evaluate the effectiveness of FN in improving patient outcomes.

**Methods:**

The Lessening the Impact of Financial Toxicity (LIFT) study is a multi-site Phase 2 clinical trial. We use a pre-/post- single-arm intervention to examine the effect of FN on FT in adults with cancer. The LIFT trial is being conducted at nine oncology care settings across North Carolina in the United States. Sites vary in geography (five rural, four non-rural), size (21–974 inpatient beds), and ownership structure (governmental, non-profit). The study will enroll 780 patients total over approximately 2 years. Eligible patients must be 18 years or older, have a confirmed cancer diagnosis (any type) within the past 5 years or be living with advanced disease, and screen positive for cancer-related financial distress. LIFT will be delivered by full- or part-time financial navigators and consists of 3 components: (1) systematic FT screening identification and comprehensive intake assessment; (2) connecting patients experiencing FT to financial support resources via trained oncology financial navigators; and (3) ongoing check-ins and electronic tracking of patients’ progress and outcomes by financial navigators. We will measure intervention effectiveness by evaluating change in FT (via the validated Comprehensive Score of Financial Toxicity, or COST instrument) (primary outcome), as well as health-related quality of life (PROMIS Global Health Questionnaire), and patient-reported delayed or forgone care due to cost. We also assess patient- and stakeholder-reported implementation and service outcomes post-intervention, including uptake, fidelity, acceptability, cost, patient-centeredness, and timeliness.

**Discussion:**

This study adds to the growing evidence on FN by evaluating its implementation and effectiveness across diverse oncology care settings.

**Trial registration:**

ClinicalTrials.gov NCT04931251. Registered on June 18, 2021.

**Supplementary Information:**

The online version contains supplementary material available at 10.1186/s13063-022-06745-4.

## Administrative information

Note: The numbers in curly brackets in this protocol refer to SPIRIT checklist item numbers. The order of the items has been modified to group similar items (see http://www.equator-network.org/reporting-guidelines/spirit-2013-statement-defining-standard-protocol-items-for-clinical-trials/).

**Table:** 

Title {1}	Lessening the Impact of Financial Toxicity (LIFT): A protocol for a multi-site, single-arm trial examining the effect of financial navigation on financial toxicity in adult patients with cancer in rural and non-rural settings
Trial registration {2a and 2b}.	NCT04931251 [ClinicalTrials.gov]
Protocol version {3}	Version 3 as of 12/1/21
Funding {4}	This research was supported by the National Cancer Institute (NCI) (1-R01-CA240092-02 and 3-P30-CA016086-44-S4, PI: Wheeler and Rosenstein). CBB is additionally supported by a NCI Cancer Care Quality Training Program grant, UNC-CH, Grant No. T32-CA-116339, for which SBW is a mentor and PI. VMP is funded by the Cancer Prevention and Control Education Program (T32CA057726-27).
Author details {5a}	Stephanie B Wheeler,^1,2^ Caitlin B. Biddell,^1,2^ Michelle L. Manning,^2^ Mindy S. Gellin,^2^ Neda R. Padilla,^2^ Lisa P. Spees,^1,2^ Cindy Rogers,^2^ Julia Rodriguez-O’Donnell,^2^ Cleo Samuel-Ryals,^1,2^ Sarah A. Birken,^3,4^ Katherine E. Reeder-Hayes,^2,5^ Victoria M. Petermann,^2,6^ Allison M. Deal,^2^ Donald L. Rosenstein^2,7^ ^1^ University of North Carolina at Chapel Hill, Gillings School of Global Public Health, Department of Health Policy and Management ^2^ University of North Carolina at Chapel Hill, Lineberger Comprehensive Cancer Center ^3^ Wake Forest School of Medicine ^4^ Wake Forest Baptist Comprehensive Cancer Center ^5^ University of North Carolina at Chapel Hill, Division of Oncology ^6^ University of North Carolina at Chapel Hill, School of Nursing ^7^ University of North Carolina at Chapel Hill, Departments of Psychiatry and Medicine
Name and contact information for the trial sponsor {5b}	Investigator initiated trial: Stephanie B. Wheeler (Co-PI) Stephanie_Wheeler@unc.edu Donald L. Rosenstein (Co-PI) donald_rosenstein@med.unc.edu
Role of sponsor {5c}	Funders do not have any role in the study design; collection, management, analysis, and interpretation of the data; writing of the report; or the decision to submit the report for publication.

## Introduction

### Background and rationale {6a}

Financial toxicity (FT), a term used to describe the multifaceted financial burden that healthcare places on patients and their families [1, 2], is a major hardship for people diagnosed with cancer, with potentially devastating effects on health and household economic outcomes [3]. This burden results from out-of-pocket *direct medical* costs (e.g., copayments, coinsurance, over-the-counter and prescription drug costs, medical supplies), as well as out-of-pocket *indirect* and *non-medical* costs related to treatment-seeking and downstream follow-up care (e.g., transportation, lost wages, lost productivity, caregiving expenses). The significant out-of-pocket *material* cost burden placed on cancer patients also frequently leads to *psychological distress* for patients and caregivers, as well as *negative coping behaviors* that affect health and health outcomes [4, 5]. Both cancer patients and caregivers report high distress and anxiety associated with cancer care costs [6, 7]. Patients with high out-of-pocket cancer care costs are more likely than those with lower out-of-pocket costs to be non-adherent to, or to delay or discontinue, recommended treatments [3]. Cancer-related financial hardship also contributes to poor health-related quality of life (HRQoL) in patients and caregivers [8, 9]. Finally, FT has been linked to more frequent bankruptcy and, in turn, bankruptcy-associated mortality [10, 11]. This increasingly prevalent hardship facing both insured and uninsured patients with cancer and their families warrants urgent intervention.

The science on FT, to date, has focused primarily on the identification of the problem, leaning on convenience sample cohorts and descriptive studies. The stage is set for the next phase of research, which includes intervention development, testing, and implementation studies of strategies to relieve the cancer-related financial burden. A variety of financial assistance programs exist and provide much-needed financial assistance to patients with cancer; indeed, the majority of NCI-designated cancer centers report screening for and providing some financial assistance support services [12]. However, these programs lack standardization, and how they are being implemented and evaluated for impact is unclear. In general, the proliferation of financial support services offered by cancer centers, nonprofit organizations, manufacturers, and the government has created a complex web of resources that, despite the most benevolent intentions, remains scattered, uncoordinated, and difficult to access, with cumbersome eligibility and paperwork requirements [13]. In addition, providers and support staff report that they lack both the time and knowledge to help patients navigate the increasingly complicated and patchwork services that do exist [13–15].

*Financial navigation (FN)* is an intervention that streamlines and coordinates access to existing financial assistance programs for patients and caregivers. FN works by systematically identifying patients at risk for FT, assessing eligibility for existing federal, nonprofit, manufacturer, and local financial support resources, clarifying treatment cost expectations, and working with patients and caregivers on an ongoing basis to develop and implement a plan to cope with high costs of care [14, 16, 17]. A financial navigator is an advocate at the helm of FN and is specially trained to work with patients and their caregivers to reduce the material and psychosocial burdens associated with the cost of cancer treatment by helping them access healthcare services, find cost-saving treatments, continue guideline-recommended care, and understand and pay their bills [18]. FN has been shown to be feasible to implement and effective in reducing cancer patients’ out-of-pocket costs by tens of thousands of dollars, reducing cost-related anxiety, and generating millions in hospital savings [17, 19, 20].

As the evidence base for FN continues to develop, attention must be paid to targeting and adapting this promising intervention to those patients at greatest risk of experiencing financial hardship and suffering poor outcomes associated with it. People living in rural areas experience both greater FT and worse cancer outcomes, on the whole, compared to non-rural patients [21–23]. Notably, the implementation context for FN is also quite different and the strategies that support more effective FN implementation in rural versus non-rural areas may also vary. However, the potential heterogeneity in FN implementation and effectiveness in rural versus non-rural oncology care settings has yet to be demonstrated; this trial examines these issues directly.

### Objectives {7}

Objectives are to (1) identify FN implementation determinants and implementation outcomes in both rural and non-rural oncology care settings and (2) evaluate the effectiveness of FN in improving patient outcomes in both rural and non-rural oncology care settings. Implementation outcomes include fidelity, uptake, acceptability, costs, and perceived sustainability. Effectiveness outcomes include improvement in patient-reported FT (primary outcome), HRQoL, and patient-reported delayed or forgone care due to cost (secondary outcomes). We hypothesize that FN, by intervening to reduce patient financial hardship, will reduce FT, improve HRQoL, and reduce delayed or forgone care due to cost. We also anticipate that implementation outcomes will support the scale-up of the intervention beyond the research setting, with unique contextual insights provided about how rural and non-rural practices can best implement and sustain FN.

### Trial design {8}

This is a prospective, multi-site, non-randomized Phase 2 clinical trial of a behavioral intervention targeting individual patients. We will use a pre- and post-FN single-arm intervention approach following a “Type II” implementation-effectiveness hybrid design with equal weight given to implementation and effectiveness outcomes. We acknowledge that a randomized, controlled design may be preferred from a statistical perspective; however, none of the participating oncology practices were interested in being randomized to a control arm, and withholding FN may have been perceived as unethical in this context; therefore, a pre-/post-FN, single-arm design was chosen.

A conceptual framework of financial burden following cancer diagnosis developed by Jones and colleagues guided the trial design and selection of outcomes [24]. In this framework, costs associated with cancer diagnosis and treatment, moderated by pre-cancer health and socioeconomic status, lead to overall financial burden, comprised of psychological (i.e., worry, ruminations) and material components (e.g., care-altering behaviors). The result of this overall financial burden, within the context of healthcare system practices (such as financial navigation), translates into patient outcomes (e.g., health-related quality of life, anxiety, and depression) [24].

## Methods: Participants, interventions, and outcomes

### Study setting {9}

The LIFT trial is underway at nine oncology practices (five rural, four non-rural) across the state of North Carolina in the United States. Sites were chosen, in part, based on their prior participation in a UNC-led statewide cancer survivorship network and the diversity of patient populations and catchments served by each North Carolina facility. Each practice setting was characterized by their rurality (according to 2010 Rural-Urban Commuting Area (RUCA) codes [25]) and other practice features. Practice settings vary in size (20–900+ inpatient beds) and ownership structure (governmental, non-profit) (Table 1). Each participating site provides care to uninsured and insured patients.

**Table 1:** Organizational characteristics of participating LIFT oncology care settings

	Overall (***N***=9)	Rural (***N***=5)	Non-rural (***N***=4)
**Cancer program type**			
NCI-designated comprehensive cancer center	1 (11%)	0 (0%)	1 (25%)
Community hospital cancer program	4 (44%)	4 (80%)	0 (0%)
Community hospital comprehensive cancer program	2 (22%)	1 (20%)	1 (25%)
Integrated cancer program	1 (11%)	0 (0%)	1 (25%)
Teaching hospital cancer program	1 (11%)	0 (0%)	1 (25%)
**Hospital ownership structure**			
Voluntary non-profit			
Private	4 (44%)	3 (60%)	1 (25%)
Other	3 (33%)	1 (20%)	2 (50%)
Government			
State	0 (0%)	0 (0%)	0 (0%)
Hospital district or authority	1 (11%)	0 (0%)	1 (25%)
Local	1 (11%)	1 (20%)	0 (0%)
**Satellite locations associated with institution**			
Yes	4 (44%)	0 (0%)	4 (100%)
No	5 (56%)	5 (100%)	0 (0%)
**Total number of staffed inpatient beds**			
0–100	2 (22%)	2 (40%)	0 (0%)
>100–200	1 (11%)	1 (20%)	0 (0%)
>200–500	3 (33%)	2 (40%)	1 (25%)
>500	3 (33%)	0 (0%)	3 (75%)
**Number of counties in catchment area**			
1	3 (33%)	3 (60%)	0 (0%)
2–10	4 (44%)	2 (40%)	2 (50%)
>10–20	1 (11%)	0 (0%)	1 (25%)
>20	1 (11%)	0 (0%)	1 (25%)

Percentages may not add up to 100% due to rounding

### Eligibility criteria {10}

The trial will enroll individuals of all gender identities who are over the age of 18 and have a confirmed cancer diagnosis within the past 5 years or are living with advanced disease. Given the prevalent and indiscriminate nature of FT among oncology patients, we will enroll all cancer types. Participants must read and speak English, plan to continue receiving care at the participating oncology study site, and be willing to complete all study activities. Participants must screen positive for cancer-related financial distress by reporting moderate to severe financial concerns, as measured using the Comprehensive Score of Financial Toxicity (COST measure) (range 0–44, ≤22 defined as eligible) [26, 27]. Patients who report mild or no financial distress will be given educational material that provides basic information about financial resources and the FN program, should they subsequently need it.

Trained financial navigators will deliver the intervention and were selected by each site among individuals meeting the following eligibility criteria: full- or part-time employee of the organization, over the age of 18, and able to read and speak English. Most sites designated individuals already serving in financial counselor or social worker roles to be trained as financial navigators. To ensure that all financial navigators are equipped to conduct the intervention, despite varying levels of experience and training, we will provide 12 h of training, discussed in further detail below.

### Who will take informed consent? {26a}

If a patient is interested and eligible, the financial navigator will present the entire informed consent document for study participation, including potential risks clearly outlined, and a HIPAA Authorization form for accessing electronic medical records. All consent procedures will follow the guidelines of the Centralized Institutional Review Board (CIRB) at the University of North Carolina at Chapel Hill. Patients will be given sufficient time to ask any questions or to take a sample consent home to consider the study options. The written consenting process will occur in a private area in the site’s cancer center or verbally by phone using the phone version of the consent. During the consenting process, it will be made clear that the patient has the right to end their study participation at any time and that their cancer care will not be affected by whether or not they participate in this study. Patients will be provided with a copy of the consent/authorization forms, which include contact information for both the Centralized Institutional Review Board and Principal Investigators (PIs) of the study. All study-related paper documents including consent forms and HIPAA forms will be stored in locked filing cabinets located in secured offices at each site. The PIs and the study coordinator will visit each site annually (if allowable due to COVID-19) to ensure that informed consent is being obtained and documents from each participant are in compliance with federal regulations and CIRB requirements.

### Additional consent provisions for collection and use of participant data and biological specimens {26b}

Not applicable—this trial will not collect participant biological specimens.

## Interventions

### Explanation for the choice of comparators {6b}

Not applicable—this is a pre-/post-FN single-arm trial with no comparison group.

### Intervention description {11a}

The FN intervention will be delivered by full- or part-time financial navigators currently employed by each oncology practice, with modest financial support provided by the grant for hiring, training, supervising, and offsetting practice-level costs associated with employing and administratively supporting staff financial navigators. Though the financial navigators are employed by each oncology practice, their role as it relates to this study is to advocate for patient financial interests. Prior to program implementation, staff participated in semi-structured interviews to determine each site’s culture (i.e., values, mission) and support of patient-centered care. Based on these interviews, we do not expect any conflict to arise for financial navigators between the site’s financial interest and the patient’s financial interest. If such a conflict does arise, however, the financial navigators’ responsibility is to advocate for the patients.

The FN intervention consists of 3 components: (1) systematic FT screening identification and comprehensive intake assessment; (2) connecting patients experiencing FT to financial support resources and assisting with application completion via trained oncology financial navigators; and (3) ongoing check-ins and electronic tracking of patients’ progress and outcomes by financial navigators. Eligible patients who consent to participate will be given the option to receive either an immediate mobile phone response with same-day point-of-care appointment scheduling or a separate scheduled appointment for a financial assessment. Both appointments will involve one-on-one consultation with the financial navigator, who reviews participants’ individual financial situation and financial assistance goals, as well as collects information about employment status, billing information, insurance status, and other indicators used to triage participants to the appropriate financial resource(s). The initial financial navigation appointment will include completion of the comprehensive intake assessment form with the financial navigator, which takes 45–60 minutes depending on the complexity of participant needs. At the conclusion of this initial appointment, participants will receive a checklist of resources they are eligible for and a list of the personal paperwork (tax forms, W-2, pay stubs) needed to apply.

At the next follow-up appointment, the financial navigator will review the initial intake forms, verify that the participant has the necessary paperwork, and consult with the participant to complete resource applications. Participants will be educated about and referred to financial resources, such as local charity care, foundation-provided financial support, medication assistance programs, Medicaid, Medicare, private health insurance plans, Social Security and Disability, and legal aid. Participants will be re-contacted by the financial navigator 2-3 weeks after each financial clinic visit to assess progress toward their financial assistance goals. The anticipated duration of intervention for participants will be between 2 weeks and 6 months, depending on needs.

The study team at UNC will employ several strategies designed to enhance the implementation of the FN intervention, including training, tailored technical assistance, peer navigator support, and external complex case management to augment local FN services, where needed. These centralized implementation strategies are designed to optimize delivery and success of the intervention and were selected based upon prior experience implementing other cancer survivorship support interventions statewide and stakeholder input.

The initial comprehensive FN training will include a modularized, interactive FN curriculum offered over a 5-week period via videoconference (due to the COVID-19 pandemic), including both synchronous and asynchronous components. The total training time over this 5-week period will be approximately 12.7 h. This training will be provided to all participating financial navigators and includes modules on cancer-related FT assessment, financial distress screening, intake processes (including the completion of study forms), descriptions of local, state, and federal financial support resources and eligibility, program evaluation metrics including participant surveys, and team-building/peer support activities. The research team will provide the synchronous training modules, including how to use Research Electronic Data Capture (REDCap), the study’s secure case management and data storage platform, and standard operating procedures (SOPs) for the study protocol, including the reporting of any adverse events. Asynchronous portions included the comprehensive Financial Advocacy Bootcamp Levels 1 and 2 are offered online by the Association of Community Cancer Centers (ACCC). This national, online resource provides basic information on federal financial aid programs and eligibility requirements, patient communication strategies, and problem-solving.

Tailored technical assistance and peer navigator support will be provided by the UNC research team. The tailored technical assistance will include monthly site-specific interactions between financial navigators at an individual site and UNC study team members, whereby specific implementation challenges can be discussed and resolved in a more targeted manner. These tailored technical assistance sessions will be delivered and audio-recorded via Zoom, with templates for meeting notes summarizing discussions derived from the Consolidated Framework for Implementation Research. Monthly group-based financial navigator support calls will be hosted and organized by the central research team and are intended to build a peer learning network and facilitate shared problem-solving. The group-based peer navigator support will include monthly teleconferences to discuss caseloads, offer best practices and lessons learned, problem-solving for difficult situations, and shared information across sites in a peer-to-peer collaborative learning format.

The Patient Advocate Foundation (PAF) will provide external complex case management to those participants for whom local navigation reached its limits—i.e., financial navigators will refer to PAF for those participants with severe and complex financial needs that extend beyond what local or general financial resources can support. In this way, we will triage participants according to the severity and complexity of their financial needs. PAF is a national 501 (c) (3) non-profit organization that, for 20 years, has provided direct financial support to patients with chronic, life threatening and debilitating diseases to help access recommended treatment [28]. PAF case managers work with patients to advocate on their behalf for access to and reimbursement for therapies, therapeutic agents, and devices deemed medically efficacious. Individual study participants will be referred to PAF directly by site-specific navigators when local or general resources have been exhausted.

### Criteria for discontinuing or modifying allocated interventions {11b}

Participants who no longer wish to participate in the intervention will be offered an educational tri-fold brochure providing basic information about financial resources and FN. Furthermore, as discussed above, the number and duration of FN follow-up appointments will be tailored to individual participant needs, and if participants wish to discontinue at any time for any reason (including that their needs have been met), they may do so.

### Strategies to improve adherence to interventions {11c}

Financial navigators will follow up with enrolled participants at the time of their oncology visits and by phone to review participant forms and work with the participant to ensure completion of all applicable resource applications. Follow-up appointments will occur every 2–3 weeks as needed to ensure that enrolled participants continue making progress toward their financial assistance goals. Financial navigators are instructed that they may reach participants via a variety of mechanisms as preferred by the participants (i.e., at visits, via text or phone call, or email).

### Relevant concomitant care permitted or prohibited during the trial {11d}

Not applicable—because this is a behavioral intervention, it is not influenced by patient care patterns. Thus, all types of cancer care are permitted during the trial.

### Provisions for post-trial care {30}

We do not expect participants to suffer harm from trial participation. The two most likely risks associated with this study include potential embarrassment, distress, or anxiety related to discussing difficult financial issues and the possible breach of confidentiality. Risk of distress or anxiety associated with financial costs of care will be directly targeted (and most likely reduced) by the intervention itself, but we acknowledge that exploring the nature of financial distress may be associated with a temporary increase in distress. We expect that both of these risks will be minimized by the careful selection and rigorous training of financial navigators (which will include an emphasis on sensitivity, privacy concerns, confidentiality, ethical behavior, maintaining dignity and respect, and creating a culturally open and non-judgmental environment in which participants feel safe from identification and free to share without harm).

### Outcomes {12}

Outcomes measured in this trial include implementation determinants, implementation outcomes, and patient outcomes (Table 2).

**Table 2:** Implementation determinants, implementation outcomes, and patient outcomes assessed in LIFT

Implementation and patient outcome measures	Data source/measurement description	Pre-FN intervention	Post-FN intervention
**Implementation determinants**			
***Financial navigator characteristics***	Financial navigator questionnaire (study-specific instrument) regarding knowledge, self-efficacy and confidence related to FT and FN	X	X
***Intervention*** complexity	Financial navigator and practice stakeholder structured interviews		X
***Outer setting*** patient needs and resources	Financial navigator and practice stakeholder structured interviews		X
***Inner setting*** implementation climate, learning climate, resources and access to information	Financial navigator and practice stakeholder structured interviews		X
***Process*** of reflecting and evaluating	Financial navigator and practice stakeholder structured interviews		X
**Implementation outcomes**			
***Fidelity*** to financial navigation protocol	Assessed via 10% random sample of all FN intake forms and appointment notes recorded by financial navigators in a patient tracking database built in RedCap to assess: whether the full comprehensive intake form was completed, whether participants received at least two visits with the financial navigator, whether the financial navigator re-contacted participants within 2–3 weeks after each financial clinic visit, and level of completeness of tracking data (e.g., were financial assistance applications submitted, was the resolution status recorded?)		X
***Uptake*** of financial navigation intervention	Assessed using patient tracking database in RedCap as the ratio of the number of patients served by FN to the number of patients screened positive for financial distress and referred to FN; and the percentage of patients who are successfully navigated to specific financial assistance resources (including, but not limited to, Social Security Disability Insurance (SSDI); Medicaid; private insurance subsidies; charity care)		X
***Acceptability*** of financial navigation to practices	Financial navigator and practice stakeholder structured interviews		X
Perceived ***sustainability*** of financial navigation	Financial navigator and practice stakeholder structured interviews		X
***Cost*** of financial navigation	Assessed using patient tracking database in RedCap and navigator time-audits as the sum of all wages associated with intervention-related navigator activity for the duration of the study period, specifically: time spent training, time spent with patients delivering financial navigation services, time spent conducting administrative functions (e.g., patient scheduling and tracking, researching financial assistance resources, and submitting and resolving financial assistance applications), and time spent engaged in technical support and tailored coaching		X
**Patient outcomes**			
Socio-demographic variables	Patient survey of age, race, ethnicity, marital status, education, income, employment status, health insurance, household size, and dependents	X	
Cancer-related financial toxicity	COmprehensive Score for financial Toxicity (COST) instrument	X	X
Emotional distress and anxiety	PROMIS Emotional Distress-Anxiety Short Form 6a: 6-item measure of anxiety over the past 7 days	X	X
Depression	PROMIS Depression Short Form 8a: 8-item measure of depression over the past 7 days	X	X
Health-related quality of life	PROMIS Global Health Questionnaire: 10-item measure of symptoms, functioning, and healthcare-related quality of life (HRQoL)	X	X
Illness impact	Psychosocial Illness Impact Short Form-Pos 4a: 4-item measure of positive emotional and social outcomes of illness	X	X
Care-altering behaviors (foregoing, delaying care)	Patient questionnaire (study-specific instrument)	X	X
Patient perspectives on FN acceptability, responsiveness and alignment with needs, patient-centeredness, and timeliness	Patient questionnaire (study-specific instrument)		X

#### Implementation determinants

Determinants of implementation success (or failure) will be evaluated using CFIR and include, briefly: intervention complexity; outer setting patient needs and resources; inner setting implementation climate, learning climate, resources and access to information; individual characteristics of knowledge, self-efficacy and confidence, and the process of reflecting and evaluating [29]. Implementation determinants will be assessed through in-depth, structured post-implementation interviews with financial navigators and other key practice-level stakeholders. A draft semi-structured interview guide is included in Additional file 1.

#### Implementation outcomes

The quality of implementation assessment will be guided by Proctor and colleagues’ implementation outcomes framework and include analysis of fidelity, uptake, acceptability, costs, and perceived sustainability [30]. Intervention acceptability and perceived sustainability will be assessed through in-depth, structured post-implementation interviews. We will measure fidelity by reviewing a 10% random sample of all FN intake forms and appointment notes recorded by financial navigators in a participant tracking database built in REDCap to assess: whether the full comprehensive intake form was completed, whether participants received at least two visits with the financial navigator, whether the financial navigator re-contacted participants within 2-3 weeks after each financial clinic visit, and level of completeness of tracking data (e.g., were financial assistance applications submitted, and was the resolution status recorded). Uptake will be assessed as the ratio of the number of participants served by FN to the number of patients screened positive for financial distress and referred to FN, and the percentage of participants who are successfully navigated to specific financial aid resources. Costs will be measured on a per-practice level as the sum of all wages associated with intervention-related navigator activity for the duration of the study period, specifically: time spent training, time spent with participants delivering financial navigation services, time spent conducting administrative functions (e.g., participant scheduling and tracking, researching financial assistance resources, and submitting and resolving financial assistance applications), and time spent engaged in technical assistance and peer support calls. These cost data will be captured through activities recorded in the participant tracking database and random navigator weekly time-audits.

#### Patient Outcomes

The primary patient outcome will be change in FT from baseline to follow-up, as measured by pre-/post-intervention completion of the Comprehensive Score for Financial Toxicity (COST) tool. The COST score was selected as the primary outcome given that it has been validated in adult patients with cancer and has been shown to be associated with relevant clinical outcomes (e.g., health-related quality of life) [27]. We will assess mean pre-/post-FN intervention COST scores, which range from 0-44 with a population mean of 22.

Secondary outcomes will include change in HRQoL and change in care-altering behavior. HRQoL will be measured using the PROMIS Global Health Questionnaire, which is a 10-item measure of symptoms, functioning, and HRQoL. We selected this secondary outcome due to the documented negative association between financial burden and HRQoL in the literature [8, 9]. We will assess differences in mean pre-/post-FN intervention HRQoL scores.

Change in care-altering behavior will be operationalized as a dichotomous outcome of forgoing or delaying care due to cost. Harmful, care-altering behavioral responses to the high cost of care, including discontinuation of and non-adherence to medications and delaying or foregoing medical treatment, may contribute to widely observed disparities in cancer mortality by rurality and socioeconomic status [1, 9, 23, 31]. We will assess mean pre-/post-FN intervention differences in the probability of forgoing or delaying care.

Additional patient outcomes will include anxiety, depression, and psychosocial illness impact, all measured using validated PROMIS scales. We will also describe frequencies, proportions, means and standard deviations, as appropriate, of patient-reported post-FN intervention convenience, accessibility, satisfaction, responsiveness (alignment with needs), patient-centeredness, and timeliness. We will use a study-specific patient questionnaire to assess these descriptive outcomes.

### Participant timeline {13}

**Fig. 1:**
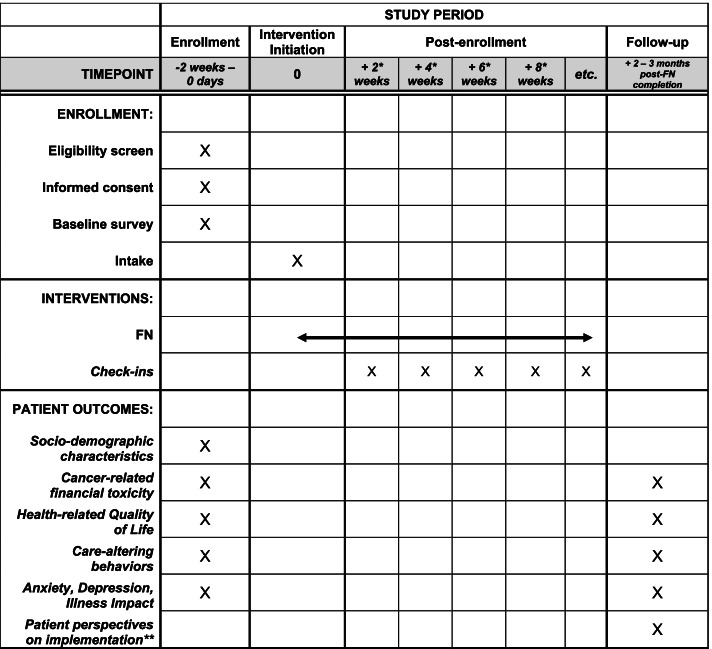
LIFT participant timeline of enrollment, intervention, and assessments. *Timing and number of follow-up visits will vary depending on each patient’s needs. ** Patient questionnaire (study-specific instrument) on FN acceptability, responsiveness and alignment with needs, patient-centeredness, and timeliness

**Fig. 2:**
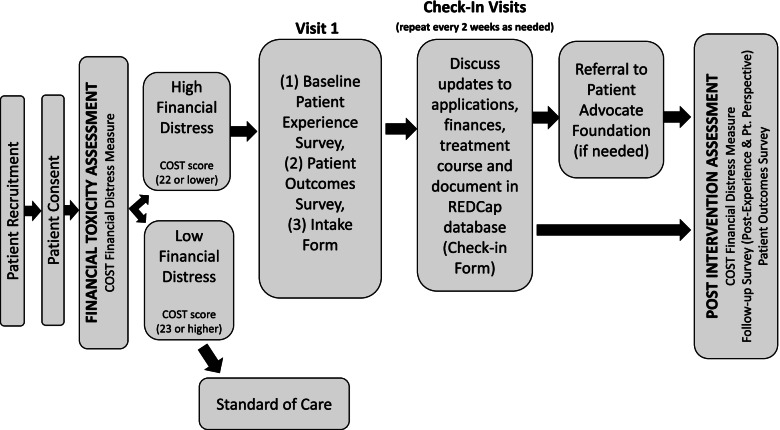
LIFT intervention flow chart

Following informed consent and baseline survey completion, participants referred to a financial navigator will have at least 2 visits (intake, follow-up), 2–3 weeks apart, with the navigator, with some participants receiving more intensive, needs-dependent support. The anticipated duration of the program for participants will be between 2 weeks and 6 months, depending on needs. Participants will be asked to complete a follow-up survey 2–3 months after intervention completion to allow time for FN to have an effect on outcomes. A schematic diagram of participant enrollment, intervention, and assessments is shown in Figs. 1 and 2 shows an accompanying flowchart of the intervention.

### Sample size {14}

We anticipate enrolling a total of 780 participants into the study in order to include 700 participants in the final analysis, allowing for attrition of up to 10%. We aim to include approximately 100 participants in the final analysis per rural site and 50 participants per non-rural site. We considered a very conservative pre/post-FN intervention effect (difference in COST scores) as small as 0.5 as well as a more considerable intervention effect as large as 3.0. A one-sample paired t-test (pre-/post- analysis of the change in COST FT score) with alpha=.05, will have 80% power to detect an effect size of 0.106. This may be seen with an average difference of 1 point, and a standard deviation of 9.43, or a mean difference of 3 points, and a standard deviation of 28.29. Notably, the UNC pilot project on which this study is based found a nearly 7-point improvement in COST FT score post-FN intervention completion.

Given our 2% (1/50) attrition rate for the UNC pilot project this study is based on [32], we do not expect much attrition. If participants are lost to follow-up between consent and their first FN visit, we will replace them since they did not receive the intervention.

### Recruitment {15}

Patients will be identified by the site staff as potentially eligible for this study based on the study eligibility criteria detailed above, and all eligibility criteria will be verified after approaching patients. Sample recruitment materials are included in Additional file 2. Recruitment of patients will be subject to established protocols defined by UNC and participating institutions. Patients will be recruited by site staff during routine clinic visits to a participating site; therefore, recruitment will not require outreach to patients who are not receiving care currently in the partner oncology sites. Each site will use a combination of passive recruitment (flyers) and active recruitment (staff introduction of the study to the patient if the patient expresses financial concerns). In most cases, staff introducing the study will be financial navigators or social workers. In both active and passive recruitment activities, it will be clear that a patient’s decision regarding participation in the study will not influence the care that they receive. In our pilot study, we were able to recruit 50 participants in four months [32]. As such, we do not anticipate challenges recruiting enough eligible participants to reach the target sample size at each site.

## Assignment of interventions: allocation

### Sequence generation {16a}

Not applicable—this is a non-randomized, single-arm intervention study.

### Concealment mechanism {16b}

Not applicable—this is a non-randomized, single-arm intervention study.

### Implementation {16c}

Not applicable—This is a non-randomized, single arm intervention study.

## Assignment of interventions: Blinding

### Who will be blinded {17a}

Not applicable—this is a non-randomized, single-arm intervention study.

### Procedure for unblinding if needed {17b}

Not applicable—this is a non-randomized, single-arm intervention study.

## Data collection and management

### Plans for assessment and collection of outcomes {18a}

After obtaining informed consent, each participant will be assigned a unique study identifier, and then proceed to complete the COST measure. If eligible (i.e., if scoring 22 or less on the COST instrument), participants will then complete the baseline patient experience survey (Additional file 3) and patient outcomes survey. Together these surveys are expected to take less than 30 minutes. Patient outcomes will be assessed using the PROMIS Global Health scale v1.2, PROMIS Emotional Distress-Anxiety – Short Form 6a v1.0, PROMIS Emotional Distress-Depression – Short Form 4a v1.0, and the PROMIS Psychosocial Illness Impact-Positive–Short Form 4a v1.0. The baseline patient experience survey will assess patients’ care-altering behaviors, employment disruption, caregiver cost burden, and the impacts of COVID-19. Following the completion of these surveys, participants will receive an appointment with the financial navigator. During the first FN appointment, financial navigators will complete a comprehensive intake form with the patient (Additional file 4). At all subsequent FN appointments, financial navigators will complete a follow-up check-in form with the patient (Additional file 5). Two to three months after the last FN visit, patients will complete the follow-up survey (Additional file 6), COST measure, and patient outcomes survey. The follow-up survey will include a post-intervention patient experience survey and participant perspectives on FN acceptability (including convenience, accessibility, and satisfaction), responsiveness and alignment with needs, patient-centeredness, and timeliness.

Each site’s financial navigator conducts all study visits with participants. A study flow diagram and study definitions intended to guide navigators through the intervention and administration of surveys and intake forms is included in Additional file 7. For maximal participant convenience and privacy, as well as data completeness, participant surveys and measurements are instructed to be administered by financial navigators via tablets at the time of financial navigation appointments, which may occur in person or via telephone calls. Participant survey data may be entered on tablets or recorded by navigators during follow-up phone calls and then entered in protocol data forms and stored in REDCap afterwards. The baseline, intake forms, check-in forms, and post-intervention participant surveys do not contain any participant identifying information and contain numeric identifiers (study IDs) instead of participant full names.

### Plans to promote participant retention and complete follow-up {18b}

Participants will receive $25 each for completing the baseline surveys and $25 each for completing the post-intervention surveys for a total of $50 per participant. Additional indirect financial incentives for study participation include the possibility of receiving benefits from financial aid resources. Participant benefits also include an improved patient care experience and potentially higher retention in oncology care as a result of taking part in the intervention and reducing financial barriers to care.

### Data management {19}

All participant data will be collected and managed via each site’s individual REDCap database, including all baseline surveys, intake forms, check-in forms, and end of study surveys. Medical record data will be abstracted by the trained financial navigator from the site’s electronic medical record and entered into REDCap as needed, such as treatment status and appointment information.

### Confidentiality {27}

The research team is committed to the confidential preservation of data and monitoring of safety of participants. All intervention tracking data and research data will be stored in REDCap, a secure data storage platform made available to UNC researchers through the North Carolina Translational and Clinical Sciences Institute. Site-specific REDCap logins prevent site staff from viewing any other site’s participant information other than their own via REDCap. Only the PIs, study coordinator, research assistant, and financial navigator who are all involved in participant recruitment and data collection will know the identity of the participants and will have access to records in the REDCap database. The PIs, study coordinator, and research assistant will be able to see enrollment in the REDCap database to ensure patient enrollment forms are complete.

To prevent a breach of confidentiality, all identifying participant information will be kept on a secure research database only accessible by sites treating those specific participants, and all de-identified research data are similarly stored on a secure research database to which only the UNC investigators have access. All investigators and site staff working on the study are CITI-certified and will be required to sign a statement promising to maintain all information strictly confidential. To conduct the intervention described above, relevant identifiable participant information will be collected upon enrollment into the study by individual site staff. These data include information such as name, date of birth, and contact information (telephone number, e-mail address). During the FN appointments, participants will provide private information to their navigator. Participants will also complete surveys at two time points during the study; however, these data are not directly linked with identifying participant information.

Data will only be transmitted via UNC's secure network and stored in our Microsoft Teams folders or transmitted among research personnel within the office environment (study meetings). Data transmitted between the server and end-users will be encrypted using SSL, and all databases will be encrypted. As stated previously, all electronic data will be kept in REDCap, which is a secure web application that is used to build and manage case report forms, surveys, and other data capture mechanisms for clinical research. REDCap has been approved by UNC as a secure location for storing sensitive research data.

### Plans for collection, laboratory evaluation, and storage of biological specimens for genetic or molecular analysis in this trial/future use {33}

Not applicable—this trial will not collect biological specimens.

## Statistical methods

### Statistical methods for primary and secondary outcomes {20a}

#### Implementation determinants and outcomes

We will use a CFIR-derived template analysis to develop themes from post-implementation interviews with navigators and stakeholders [33]. We will produce practice-specific and general summaries of findings in a matrix format, following prior work [29]. In addition, we will use standardized barrier/facilitator ratings (ranging from −2 to +2) based upon coded interview data to score the extent to which CFIR-derived constructs were viewed as impediments or accelerants to FN implementation. We will assess and describe FN fidelity using a checklist and the range in uptake and costs across practices.

#### Patient outcomes

We will assess mean pre-/post-FN intervention FT (operationalized as COST scores) using a paired two-sided sample t-test to test the null hypothesis that FN does not result in statistically significant changes in FT, at an alpha level = 0.05. We will again use a paired two-sided sample *t*-test to test the null hypothesis that FN does not result in statistically significant changes in HRQoL, at an alpha level = 0.05. We will use a paired McNemar chi-squared test to test the null hypothesis that the probability of foregoing or delaying care is reduced post-FN is the same as the probability that foregoing or delaying care is increased post-FN.

### Interim analyses {21b}

We will conduct preliminary analyses at 100 patients enrolled and 200 patients enrolled to ensure data completeness and to understand intervention implementation.

### Methods for additional analyses (e.g., subgroup analyses) {20b}

We will assess geographic (rural vs. non-rural) differences in our outcomes, and where feasible (depending on sample sizes within subgroups), racial/ethnic, gender, and age-specific effect modification for the main participant outcome of pre/post-FN change in FT.

### Methods in analysis to handle protocol non-adherence and any statistical methods to handle missing data {20c}

We plan to compare characteristics between participants who do and do not complete follow-up measures and report on any differences to inform additional analyses. Because final primary outcome analyses must compare pre/post differences in FT among participants completing both the baseline and follow-up surveys, we will exclude participants who are missing follow-up survey data.

### Plans to give access to the full protocol, participant-level data and statistical code {31c}

Given the sensitive nature of the participant financial and clinical data to be collected in this trial, public access to participant-level data will not be permitted. However, upon request, data-sharing agreements to analyze de-identified, aggregated patient-level data will be permitted (discussed in further detail in “Availability of data and materials” section). Statistical code and additional information about the protocol may be requested from the UNC research team.

## Oversight and monitoring

### Composition of the coordinating center and trial steering committee {5d}

The UNC IRB is the central IRB (CIRB) of record for this study. We have completed all reliance paperwork required and, as part of the IRB application Multi-Site Study Information, Federal Assurance numbers were collected for all sites that have them. Each site has a designated Signatory Official and Contact person. In addition, the study has an External Advisory Board (EAB) that meets annually to provide expertise on rural health, dissemination and implementation of evidence-based interventions, qualitative methods, patient-centered cancer treatment decision-making, and cancer-related financial hardship. At the site level, oversight and monitoring occur monthly through efforts described previously.

### Composition of the data monitoring committee, its role, and reporting structure {21a}

As stated in the Further Guidance on a Data and Safety Monitoring for Phase I and Phase II Trials, an independent Data Safety and Monitoring Board may not be necessary or appropriate when the intervention is low risk, as is the case in this study. In this single-arm/non-randomized, Phase 2 clinical trial of a behavioral intervention with a very low risk for severe and unacceptable side effects, we follow NIH guidance and opted not to appoint a Data Safety and Monitoring Board. The PIs will provide continuous monitoring of patient safety in this trial.

Meetings/teleconferences will be held at a frequency dependent on study accrual, and in consultation with the study Biostatistician. At these meetings, the research team will discuss all issues relevant to study progress, including enrollment, safety, regulatory, data collection, etc. and the team will produce summaries or minutes of these meetings. These summaries will be available for inspection when requested by any of the regulatory bodies charged with the safety of human subjects and the integrity of data including, but not limited to, the oversight (Office of Human Research Ethics (OHRE) Biomedical IRB, the Oncology Protocol Review Committee (PRC) or the North Carolina TraCS Institute Data and Safety Monitoring Board (DSMB).

### Adverse event reporting and harms {22}

In the event that an unanticipated problem occurs at a site, the financial navigator will notify the PI at their site of the problem and appropriate referrals will be made to resolve the issue. The PI at the site will let the UNC PIs and the project manager know about the issue if appropriate. The project manager will log the event and share information with all sites within 24 h for severe complaints if appropriate.

### Frequency and plans for auditing trial conduct {23}

Each site will participate in two monthly video conferences and/or telephone calls (i.e., tailored technical assistance, group-based peer navigator support), and one annual in-person site visit with the UNC data management team to discuss accrual, retention, and compliance with forms. As an investigator-initiated study, this study will also be monitored generally by the Lineberger Comprehensive Cancer Center Data Safety and Monitoring Committee every twelve months.

### Plans for communicating important protocol amendments to relevant parties (e.g., trial participants, ethical committees) {25}

The UNC study coordinator will act as the liaison between participating institutions, the study, the PIs, and the CIRB. This person will be responsible for all protocol-related correspondence with the CIRB, and for relaying any official communications from the CIRB to the study team. If necessary, the project leads at each study site will communicate and relay information and documents between their local IRB and the CIRB.

The study coordinator will ensure that each site’s designated PI and financial navigator, receives a copy of the current study protocol, protocol amendments, and any other necessary study-related communication. Specifically, the study coordinator will send each site a copy of all protocol amendments within 7 days of approval of protocol changes and sites will be cc’d on study-related communications impacting their site.

### Dissemination plans {31a}

We understand and agree to comply with the NIH policy on Dissemination of Clinical Trial Information. The PIs and co-investigators acknowledge their willingness to share data and results with other eligible investigators through academically established means.

The clinical trial is posted on ClinicalTrials.gov, and the informed consent documents for this clinical trial will include a specific statement regarding the posting of trial information to the ClinicalTrials.gov website. Additionally, in concordance with NIH policy, the University of North Carolina at Chapel Hill is a registered Sponsor on ClinicalTrials.gov, and the UNC Office of Clinical Trials has named a Protocol Registration and Results System administrator to oversee registrations.

At study completion, we will disseminate our results via scientific journals and presentations, as well as through data briefs and other formats via the National Cancer Institute, the Cancer Prevention and Control Research Network (of which Dr. Wheeler is Coordinating Center PI), the American Cancer Society and Susan G Komen Foundation (through which Drs. Reeder-Hayes and Wheeler have ongoing partnerships), Livestrong, the American Psycho-Oncology Society, and the Association of Oncology Social Workers (through which Dr. Rosenstein has ongoing partnerships). Additionally, we will leverage our existing statewide network of oncology practices and an expert advisory board to create and refine strategies to promote the implementation and effectiveness of FN to be disseminated to practices beyond the study context.

## Discussion

The LIFT trial was designed as an individualized, pragmatic, and scalable intervention for cancer patients experiencing FT. It is anticipated that this study will inform and improve the implementation of financial navigation by identifying the critical components and optimum practices associated with addressing the financial needs of cancer patients cared for in both rural and non-rural oncology practices. LIFT seeks to equip stakeholders from various healthcare systems, payers, and financial assistance programs with tools to better coordinate and promote access to financial assistance supportive care and can be delivered flexibly and remotely outside the clinical encounter. Findings from this trial will add to the rapidly developing literature on financial navigation by generating evidence related to its context-specific implementation and effectiveness in diverse oncology practices across the state of North Carolina.

The LIFT protocol should be considered in the context of several limitations. Despite enrolling participants from nine oncology practices representing diverse geographies, sizes, and practice ownership structures, LIFT is limited, at this point, to practices and patients within the state of North Carolina. As such, findings may not be generalizable to the implementation of FN in other states. In this regard, LIFT may have more direct relevance for FN delivered in other states that, like North Carolina, have not expanded Medicaid coverage. As noted above, LIFT was designed as a single-arm study with a pre-post intervention design. As such, this trial will not address the efficacy of LIFT compared to usual care; instead, this trial will address within-person improvement in FT post-program completion. Further, FN, by nature, is tailored to individual patient needs. Consequently, the intensity, length, and specific FN recommendations will vary by patient, which may influence the intervention’s effectiveness in different settings. Finally, FT is not a static problem. The constantly changing nature of health care system ownership, management practices, insurance premiums, and co-pays, coupled with the fluctuating funding capacity of non-profit organizations, underscores the challenges of any intervention targeting an inherently unstable problem.

Despite these limitations, we believe the LIFT trial, by directly and flexibly addressing the financial needs of patients across diverse practice settings in North Carolina, has the potential to significantly contribute to the evidence base on addressing financial hardship experienced by patients with cancer and enhancing the implementation and sustainability of such interventions. This work builds on several related initiatives conducted by our multidisciplinary study team, including a pilot FN study at North Carolina Cancer Hospital and partnerships with a UNC-led statewide survivorship support network [32]. As such, we are confident in our ability to meet our recruitment targets and complete LIFT as described. The impact of this work will be to provide actionable and measurable recommendations about implementing FN programs in diverse oncology setting to reduce patients’ FT, diminish rural-urban, socioeconomic, and racial/ethnic disparities in cancer care access, and improve coordination and outcomes of care.

## Trial status

The trial was registered on ClinicalTrials.gov on June 18, 2021, as protocol NCT04931251. Study procedures were approved by the University of North Carolina at Chapel Hill’s Institutional Review Board (IRB-20-1997, IRB-20-3181, IRB-19-3156) on March 3, 2021. Recruitment began on November 12, 2021, and will continue through approximately March 2023.

## Supplementary Information

**Additional file 1.** Post-Implementation Interview Guide – includes a draft of the semi-structured interview guide to be used following intervention implementation

**Additional file 2.** Recruitment Materials – includes a screenshot of the LIFT webpage and the flyer given to patients for recruitment

**Additional file 3.** Baseline Survey – includes the patient experience survey given to patients prior to the intervention

**Additional file 4.** Intake Form – includes the form used by navigators to assess patient financial needs and potential resources

**Additional file 5.** Check-in Form – includes the form used by navigators to follow-up on patient financial needs and receipt of resources throughout the study

**Additional file 6.** Follow-up Survey – includes the patient experience survey given to patients following the intervention and a patient perspective survey about the intervention

**Additional file 7.** Navigator Materials– includes a study schema designed to help navigators through the study process and a table of definitions given to navigators to ensure consistency and understanding across sites

## Data Availability

We endorse the principle that resource and data sharing is essential for the accelerated translation of clinical and translational research along the continuum from discovery to improvement in human health. Thus, we commit to broadly sharing appropriate materials and data that are developed with support from the NIH, while protecting participant privacy and confidentiality in accordance with best human subjects research practices and Health Insurance Portability and Accountability Act (HIPAA) guidelines. We expect that our study will generate quantitative data at the levels of individual patients and clinical sites. We will work with our REDCap data management developers and statistical partners to de-identify the data collected in accordance with the standards set forth by HHS Regulations for the Protection of Human Subjects and HIPAA requirements before making data publicly available. In order to reduce the risk of deductive disclosure, we will not share site-level data given the rural and small geographic regions where the clinics are located. However, we will plan to share the aggregate data set (*n*=700) which includes data from all 9 clinical sites. Upon request, data-sharing agreements to analyze de-identified data will be created, reviewed for compliance, and signed by all relevant parties. In the data-sharing agreement, outside researchers must commit to only using the data for research purposes; not manipulating the data to try to identify individual patients or sites; and commit to destroying the data after analyses are complete. The UNC PIs of this study will manage both data usage requests and subsequent data-sharing agreements.

## Abbreviations

CFIR: Consolidated Framework for Implementation Research
CIRB: Central Institutional Review Board
COST: Comprehensive Score for Financial Toxicity
FN: Financial navigation
FT: Financial toxicity
HRQoL: Health-related quality of life
IRB: Institutional Review Board
LIFT: Lessening the Impact of Financial Toxicity
PIs: Principal Investigators
REDCap: Research Electronic Data Capture
UNC: University of North Carolina at Chapel Hill
