# Supplementary material for: Lessening the Impact of Financial Toxicity (LIFT): a protocol for a multi-site, single-arm trial examining the effect of financial navigation on financial toxicity in adult patients with cancer in rural and non-rural settings
Source: Trials. 2022 Oct 3;23:839. doi: 10.1186/s13063-022-06745-4 (PMC9527389; doi:10.1186/s13063-022-06745-4)
Supplement: Supplementary file 1 — Additional file 1. Post-Implementation Interview Guide – includes a draft of the semi-structured interview guide to be used following intervention implementation [file 13063_2022_6745_MOESM1_ESM.pdf]

## Aim 2 Community-Based Oncology Practice Key Stakeholder
## Semi-Structured Interview Guide

45 minutes to 1 hour

**Introduction:**

Thank you for your interest in this study. Thanks so much for completing the online survey we sent prior to this interview. The aim of this interview is to help us understand your practice's experience implementing the LIFT intervention. Your responses to both the survey and the interview will help us understand how to improve cancer programs' ability to implement LIFT moving forward.

We expect that our discussion will last about 45 minutes to an hour. Are you in a place where you feel comfortable and like you can speak freely, i.e., in an office with a door that closes? Everything you tell us will remain confidential and will only be reported as part of a bigger group, without your name attached to it. Before we begin, I would like to ask your permission to audio record our discussion (for research and training purposes). Would it be OK with you if I record this interview? (*If participant refuses to be audio-recorded, the project coordinator will take notes instead*) The interview will be turned into written notes, but your name or any identifying details will not be associated with any of the notes. The audio recordings will be erased once the project is complete.

Do you have any questions before we begin?

If you have any questions, please reach out to the study staff or the PI for this study [EMAIL; PHONE#].

**1.** Role in the organization

**2.** I'd like to start by asking you to describe how LIFT implementation went in your organization. Was LIFT used in the way that the training indicated it should be? Was it used for all eligible patients? Did all of the staff and providers who were supposed to use LIFT do so? Was LIFT used for all eligible patients?

**3.** Now I'd like to ask you about how people in your organization perceived LIFT. Were people in your organization satisfied with LIFT? Consider both the intervention itself as well as the strategies that you were offered to facilitate LIFT's implementation (i.e., training [including ACCC Financial Advocacy Bootcamp], technical support, tailored coaching).

**4.** Did people in your organization perceive LIFT as compatible with the organization (e.g., its mission, values, existing systems)?

**5.** Do people in your organization perceive LIFT as something that could be used once the study is over? Consider both the intervention itself as well as the strategies that you were offered to facilitate LIFT's implementation (i.e., training [including ACCC Financial Advocacy Bootcamp], technical support, tailored coaching).

**6.** Now, I'd like to **discuss barriers and facilitators** to implementing LIFT in your organization.

The LIFT program consists of: (1) identification of cancer patients at high risk for, or currently experiencing financial difficulties related to their cancer treatment; (2) connecting these patients to a dedicated oncology financial navigator in your organization (supported by a UNC grant in this context), who will use a comprehensive assessment tool to determine financial needs and one-on-

one appointments to direct patients to specific financial support resources and assist with applications; and (3) routine tracking and monitoring of patients' financial and health outcomes. The patients referred to a financial navigator have at least 2 visits with the navigator with some patients receiving more intensive, needs-dependent support.

When I say "LIFT implementation," I am referring to people in your organization using LIFT correctly for all eligible patients.

Could you please talk about how implementing LIFT in your organization worked?

Were there things facilitated LIFT's implementation?

Were there things that made implementing LIFT challenging?

**Prompts – potential LIFT implementation determinants**

| | |
|---|---|
| **CFIR: OUTER SETTING** | |
| ***POPULATION*** | (How) Did your patients' needs influence LIFT implementation? |
| ***COSMOPOLITANISM*** | Did any exchange information of with others outside of your organization influence LIFT implementation?<br><br>What professional networking do you engage in? Local or national conferences? Social media? |
| ***PEER PRESSURE*** | Did you feel pressure from other organizations to implement LIFT or not to implement lift? |
| ***EXTERNAL POLICY & INCENTIVES*** | Are there any local or national guidelines that play a role in whether or not/how you implemented LIFT?<br><br>Are there financial incentives provided by your practice or another organization that would influence whether or not/how you implemented LIFT? |
| **CFIR: PROCESS** | |
| ***ENGAGING*** | Are there key influential individuals that would affect whether/how LIFT was implemented? |
| **CFIR INNER SETTING** | |
| ***STRUCTURAL CHARACTERISTICS*** | Do you think the infrastructure of your organization (social architecture, age, maturity, size, or physical layout) affected LIFT implementation? |
| ***NETWORKS & COMMUNICATION*** | How do you typically find out about new information within your organization/practice about how/whether to implement LIFT?<br>• How would information about how to implement LIFT be shared in your practice? |
| ***CULTURE*** | Are there any aspects of your organization's culture (general beliefs, values, assumptions that people embrace) that affected whether or not/how LIFT was implemented in your organization? |
| ***IMPLEMENTATION CLIMATE*** | Do you think that there is a strong need to implement LIFT in your practice?<br><br>Are there standard work processes and practices regarding LIFT implementation in your practice?<br><br>Did implementing LIFT conflict with other priorities in your organization? |
| ***CFIR: INDIVIDUAL CHARACTERISTICS / TDF DOMAINS*** | |

| | |
|---|---|
| ***KNOWLEDGE (TDF)/ KNOWLEDGE AND BELIEFS ABOUT THE INTERVENTION (CFIR)*** | Are there guidelines or rules that make recommendations regarding LIFT implementation?<br><br>What was the role, if any, of these guidelines or rules in LIFT implementation? |
| ***BELIEFS ABOUT CAPABILITIES (TDF)/ SELF-EFFICACY (CFIR)*** | How confident were you in issuesLIFT implementation? (*Prompts: problems you may encounter/additional expertise or experience needed*) |
| ***BELIEFS ABOUT CONSEQUENCES (TDF)*** | What were the potential benefits or disadvantages to you to <u>not</u> implementing LIFT? |
| ***MOTIVATION & GOALS (TDF)*** | How/was it important  to you <u>to implement LIFT</u>? |
| ***MEMORY, ATTENTION, AND DECISION PROCESSES (TDF)*** | What, if any, were the tasks that you do automatically when faced with implementing LIFT? (*Prompts*: Would there be conditions under which you would consider helping patients deal with financial issues an automatic task?) |
| ***ENVIRONMENTAL CONTEXT AND RESOURCES (TDF)*** | What physical or environmental resources influenced whether/how you implemented LIFT? |
| ***SOCIAL INFLUENCES (TDF)/ INDIVIDUAL IDENTIFICATION WITH THE ORGANIZATION (CFIR)*** | (How) Did views or opinions of others, such as colleagues, patients, professional groups, or others in your practice influence whether/how you implemented LIFT?<br><br>Is there consensus in the profession about whether/how LIFT should be implemented? |
| ***EMOTION (TDF)*** | What role, if any, did your emotions or emotions of others (e.g., frustration, indifference) influenced whether or not/how LIFT was implemented in your organization? |
| ***BEHAVIORAL REGULATION (TDF)*** | What do you think would have to change at your practice to implement LIFT? |

Thank you so much for talking with us.
