# Supplementary material for: Lessening the Impact of Financial Toxicity (LIFT): a protocol for a multi-site, single-arm trial examining the effect of financial navigation on financial toxicity in adult patients with cancer in rural and non-rural settings
Source: Trials. 2022 Oct 3;23:839. doi: 10.1186/s13063-022-06745-4 (PMC9527389; doi:10.1186/s13063-022-06745-4)
Supplement: Supplementary file 2 — Additional file 2. Recruitment Materials – includes a screenshot of the LIFT webpage and the flyer given to patients for recruitment [file 13063_2022_6745_MOESM2_ESM.pdf]

LIFT Recruitment Materials

Website (cancercosts.org)

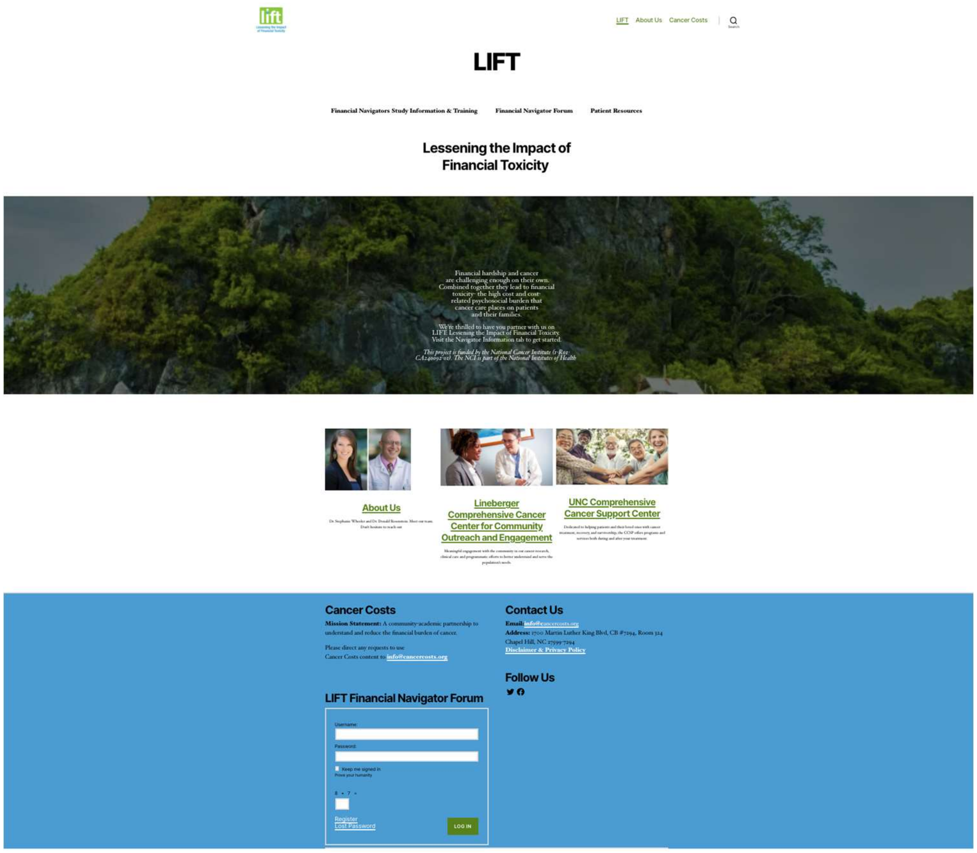

Patient Flyer – *shown on next page*

Lessening the Impact of Financial Toxicity

Looking for help with your finances ($) because of cancer?

Cancer can be hard enough. But when you have money concerns caused by cancer, it is even harder. We want to help and we are interested in learning whether having additional help over a period of time can help reduce the stress and burden caused by the cost of cancer and cancer care.

If this sounds appealing, please read below.

WHO: If you are a cancer patient or survivor who has been diagnosed within the last 5 years and/or living with advanced disease.

WHAT: Join our research study to ***receive help finding financial support services*** (called financial navigation) and ***help us learn*** how financial navigation can reduce the stress and burden caused by cancer.

There is no cost to you and you will receive $50.00 for completing this research study. (*After you complete the first surveys you will receive a $25 gift card. When you finish participating, you will complete follow-up surveys and receive another $25 gift card.*)

WHERE: Visits with financial support navigators may occur in-person or on the phone. You will complete 1 to 4 visits depending on your needs. Surveys may be completed online or on paper, depending on your preference.

WHEN: If your visits are in-person, we will try to meet with you while you're already here, or a time convenient to you. If your visits are on the phone, we will schedule a time that works for you!

HOW: Contact [NAME] at [PHONE/ EMAIL] for more information.

All information will be confidential. We look forward to hearing from you!

UNC LINEBERGER COMPREHENSIVE CANCER CENTER

SITE LOGO

LIFT Quick Study Reference Guide 2021-2023: This research is funded by the National Cancer Institute at the National Institutes of Health (grant # 5R01CA240092-02) and (grant# 5P30CA016086-44). It has been reviewed and approved by the University of North Carolina-Chapel Hill: IRB Study # 20-1997
