# Supplementary material for: Lessening the Impact of Financial Toxicity (LIFT): a protocol for a multi-site, single-arm trial examining the effect of financial navigation on financial toxicity in adult patients with cancer in rural and non-rural settings
Source: Trials. 2022 Oct 3;23:839. doi: 10.1186/s13063-022-06745-4 (PMC9527389; doi:10.1186/s13063-022-06745-4)
Supplement: Supplementary file 3 — Additional file 3. Baseline Survey – includes the patient experience survey given to patients prior to the intervention [file 13063_2022_6745_MOESM3_ESM.pdf]

Study ID: ___________ **Study Site**: ____________ **Date**: ____________ **FN**: ___________

PRE Patient Experience Questionnaire

Please answer the following questions so that we can better understand your needs as a patient with cancer. Your answers to these questions will help us learn what concerns you most about your financial situation and ability to get healthcare so we can help you get other support if it is available. Please feel free to ask (your LIFT financial navigator) any questions you have about this survey.

THIS SET OF QUESTIONS ASKS ABOUT YOUR FINANCIAL SITUATION BEFORE BEING DIAGNOSED WITH CANCER:

1. **During the 5 years before your diagnosis, who did you usually see when you were sick or needed advice about your health? Choose one:**

- ☐ General practitioner, internal medicine or family doctor, physician's assistant (PA) or nurse practitioner (NP)
- ☐ Specialist such as heart, lung, or kidney doctor
- ☐ Emergency room or urgent care doctor
- ☐ Local health department
- ☐ Did not have a health care provider
- ☐ Other, specify: ______________________________

2. **During the 5 years before your diagnosis, did you:**

See a regular doctor for a physical examination or check-up?	☐ Yes	☐ No
Have an eye exam to check your eye sight?	☐ Yes	☐ No
See a dentist or dental hygienist to check your teeth?	☐ Yes	☐ No
Have a Flu Vaccine?	☐ Yes	☐ No
Have a colonoscopy or sigmoidoscopy, that is, an examination with a lighted tube to check for signs of cancer in your rectum or colon?	☐ Yes	☐ No
Have a pap smear or pap test, that is, an examination to check for signs of cancer in your cervix?	☐ Yes	☐ No
Have a mammogram, that is, an examination with an x-ray image to check for signs of cancer in your breasts?	☐ Yes	☐ No

THIS SET OF QUESTIONS ASKS ABOUT YOUR FINANCIAL SITUATION SINCE BEING DIAGNOSED WITH CANCER:

3. At the time of your diagnosis, did you have health insurance?

☐ Yes
☐ No (**Skip** to 4)

3a. (IF YES) At the time of your diagnosis, what type(s) of health insurance did you have? Select all that apply:

☐ Private health insurance through your employer or your partner's employer
☐ Private health insurance bought by yourself or your partner, such as through a healthcare market exchange
☐ Medicaid
☐ Medicare
☐ Tricare or other military health insurance, including VA healthcare
☐ Any other insurance that covers part of your medical bills, please specify:

____

4. Over the past few months have you stayed in a job because you were concerned about losing your health insurance?

☐ Yes
☐ No
☐ N/A (e.g., have not had a job since diagnosis)

5. Since your diagnosis, what out-of-pocket costs (i.e., costs to you that insurance doesn't cover) have you or your family had related to your health? (Please mark all that apply).

☐ Medications (e.g., prescription drugs and over-the-counter medications)
Note: *Only answer 5a, if you checked Medications*
5a. Was this cost expected or unexpected?
☐ Expected
☐ Unexpected

☐ Home healthcare (e.g., nursing, home medical supplies)
Note: *Only answer 5b, if you checked Home healthcare*
5b. Was this cost expected or unexpected?
☐ Expected
☐ Unexpected

☐ Counseling services (e.g., therapy, grief counseling)
Note: *Only answer 5c, if you checked Counseling services*
5c. Was this cost expected or unexpected?
☐ Expected
☐ Unexpected

☐ Rehabilitation therapies (e.g., occupational, speech, or physical therapy)
Note: *Only answer 5d, if you checked Rehabilitation therapies*
5d. Was this cost expected or unexpected?
☐ Expected
☐ Unexpected

☐ Alternative therapies (e.g., acupuncture, massage, hypnosis)
Note: *Only answer 5e, if you checked Alternative therapies*
5e. Was this cost expected or unexpected?
☐ Expected
☐ Unexpected

☐ Other healthcare costs (e.g., copays for office or ER visits, additional referrals, imaging and lab tests)
Note: *Only answer 5f, you checked Other health care costs*
5f. Was this cost expected or unexpected?
☐ Expected
☐ Unexpected

☐ Lodging associated with your healthcare (e.g., hotel costs)
Note: *Only answer 5g, if you checked Lodging*
5g. Was this cost expected or unexpected?
☐ Expected
☐ Unexpected

☐ Transportation associated with your healthcare (e.g., parking, gas)
Note: *Only answer 5h, if you checked Transportation*
5h. Was this cost expected or unexpected?
☐ Expected
☐ Unexpected

☐ Diet (e.g., enteral feeds, formula)
Note: *Only answer 5i, if you checked Diet*
5i. Was this cost expected or unexpected?
☐ Expected
☐ Unexpected

☐ I have not had any out of pocket costs related to my healthcare.

☐ Other:____

Note: Only answer 5j, if you checked Other

5j. Was this cost expected or unexpected?

☐ Expected

☐ Unexpected

6. **Have you <u>ever</u> stopped or refused a treatment related to your cancer because it cost too much?**

☐ Yes

☐ No

7. **Have you <u>ever</u> been contacted by a debt collection agency as a result of your cancer care?**

☐ Yes

☐ No

8. **Which of the following have you <u>ever</u> done to manage the cost of your cancer care? Select all that apply**

Skipped a vacation or other activity due to cost	☐
Borrowed money from friends, family, a bank, or other source	☐
Taken money from a savings or retirement account	☐
Filed for bankruptcy	☐
Avoided treatment for another medical problem (other than your cancer)	☐
Skipped paying non-medical bills like rent, credit cards, or other necessities	☐
Reduced spending on basics like food or clothing	☐
Sold stocks or investments	☐
Mortgaged, refinanced, or sold home	☐
Moved or downsized your home	☐

9. **<u>Since your diagnosis</u>, how often did you do the following due to cost?**

	**Never**	**Rarely**	**Sometimes**	**Often**
Delayed filling your prescribed medication	☐	☐	☐	☐
Filled only part of your prescribed medication	☐	☐	☐	☐
Avoided making your clinic visits	☐	☐	☐	☐
Decided not to use medically-related services such as physical therapy	☐	☐	☐	☐
Decided not to have a medical test performed	☐	☐	☐	☐
Decided not to receive other non-cancer preventive care (such as flu shots, cancer screenings, dental cleanings)	☐	☐	☐	☐

10. **Since your diagnosis, how often did you not see a medical provider because of transportation issues?**
    - o Never
    - o Rarely
    - o Sometimes
    - o Often

11. **Since your diagnosis, how often have you been worried about the cost of travel (e.g., miles, gas) or lodging (e.g., hotel, apartment) related to your cancer care?**
    - o Never
    - o Rarely
    - o Sometimes
    - o Often

12. **Since your diagnosis, how much have you worried about how the cost of your cancer care has impacted your loved ones?**
    - o Not at all
    - o Some/a little
    - o A lot
    - o All the time/constantly

13. **Within the past 12 months, how often have you worried whether your food would run out before you got money to buy more?**
    - o Often True
    - o Sometimes True
    - o Never True
    - o Don't Know/Refused

14. **Within the past 12 months, how often did the food you bought just not last and you didn't have money to get more?**
    - o Often True
    - o Sometimes True
    - o Never True
    - o Don't Know/Refused

THE NEXT SET OF QUESTIONS ASKS ABOUT YOUR HOUSEHOLD EMPLOYMENT AND INCOME:

15. **Which best describes your job(s)?**

If you have more than one job, please check all that apply. If you are not working, please tell us about the last job you had.

- ○ **Sales, technical, or administrative support** (for example, medical assistant, real estate agent, store manager, administrative assistant, sales associate)
- ○ **Service provider** (for example housekeeper, home attendant, police officer, food server, janitor, hair-dresser, aesthetician)
- ○ **Operator, fabricator, or laborer** (for example utility worker, driver, seamstress/tailor, factory worker, laundry worker)
- ○ **Managerial or professional specialty** (for example, accountant, computer specialist, attorney, teacher, health practitioner)
- ○ **Arts, media or athletics** (for example artist, entertainer, journalist)
- ○ **Other (please specify): ________________________**

16. **Which would you say best describes your workplace(s)?**

If you have more than one job, please check all that apply. If you are not working, please tell us about the last job you had.

- ○ **Service** (for example restaurant, hotel, housekeeping, homecare provider, tailor, laundry)
- ○ **Retail** (for example a shop or store)
- ○ **Government** (for example post-office, courts, police or fire department)
- ○ **Manufacturing** (for example factory worker)
- ○ **Finance, insurance, or real estate**
- ○ **Wholesale Trade**
- ○ **Transportation and public utilities**
- ○ **Construction**
- ○ **Healthcare/Medical**
- ○ **Legal**
- ○ **Education**
- ○ **Arts and Entertainment**
- ○ **Other** (please specify): __________

**17**	What was your employment status at the time of your cancer diagnosis?	☐ Employed full time<br>☐ Employed part time<br>☐ Not employed, but looking for work, *skip to #25*<br>☐ Not employed – not retired, not disabled, and not looking for work, *skip to #25*<br>☐ Retired, *skip to #25*<br>☐ Disabled, not able to work, *skip to #25*<br>☐ Other, please specify: ______ , *skip to #25*

**18**	Has your cancer diagnosis led to any of the following changes in your employment? Please mark all that apply.	☐ Retired early<br>☐ Left my job<br>☐ Decreased work hours<br>☐ Increased work hours<br>☐ Briefly changed work schedule to attend medical appointments<br>☐ Changed job or careers<br>☐ No change to employment<br>☐ Other, please specify:______
**19**	Did you miss any days of work because of your cancer diagnosis?	☐ Yes<br>☐ No, *skip to #21*
**20**	Did you use any of the following for absences from work due to your cancer diagnosis? Please mark all that apply.	☐ Family Medical Leave Act (FMLA)?<br>☐ Paid sick leave<br>☐ Paid vacation leave<br>☐ Unpaid leave<br>☐ None<br>☐ Other, please specify:______
**21**	Did your doctor or a member of the healthcare team ever have a conversation with you about the impact that cancer may have on your ability to stay employed?	☐ Yes<br>☐ No<br>☐ Not sure
**22**	Did you speak to your employer or Human Resources (HR) department about how cancer may impact your ability to do your job?	☐ Yes<br>☐ No<br>☐ Not sure
**23**	Are there ways that your employer helped you after you were diagnosed with cancer? Please mark all that apply.	☐ Changed job duties/responsibilities<br>☐ Allowed additional paid time off<br>☐ Offered assistance that helped cover medical and non-medical bills<br>☐ Offered assistance in understanding insurance benefits and coverage<br>☐ Discussed different leave options<br>☐ Provided option to work from home<br>☐ Provided flexible work schedule<br>☐ Other (please specify:_____)

**24**	Are there ways that your employer could have better supported you since you were diagnosed with cancer? Please mark all that apply.	☐ Changed job duties/responsibilities<br>☐ Allowed additional paid time off<br>☐ Offered assistance that helped cover medical and non-medical bills<br>☐ Offered assistance in understanding insurance benefits and coverage<br>☐ Discussed different leave options<br>☐ Provided option to work from home<br>☐ Provided flexible work schedule<br>☐ Other (please specify:_____)

THE NEXT SET OF QUESTIONS ASKS ABOUT YOUR CURRENT FINANCIAL SITUATION:

25. How confident are you that you could find the money to pay for a *financial emergency* that costs about $1000?

1	2	3	4	5	6	7	8	9	10

No Confidence — Little Confidence — Some Confidence — High Confidence

26. How often do you find yourself "just getting by" financially or living *paycheck to paycheck?*

1	2	3	4	5	6	7	8	9	10

Never — Sometimes — Often — Always

27. How hard is it for you to pay for the very basics like food, housing, medical care, and heating?

1	2	3	4	5	6	7	8	9	10

Never Hard — Sometimes Hard — Often Hard — Always Hard

THIS FINAL SET OF QUESTIONS ASKS ABOUT HOW THE NOVEL CORONAVIRUS DISEASE (ALSO KNOWN AS COVID-19) HAS AFFECTED YOUR CANCER CARE AND FINANCES:

28. Has there ever been a time you had to delay or stop your cancer treatment because of the coronavirus/COVID-19 pandemic?

☐ Yes

☐ No

29. Have you had any of the following healthcare services delayed or canceled because of the coronavirus/COVID-19 pandemic?

☐ Surveillance monitoring (cancer monitoring)
☐ Lab tests
☐ Scans/imaging
☐ Cancer-related surgery
☐ Chemotherapy or other cancer-related infusion appointments
☐ Radiation therapy appointments
☐ Cancer-related follow-up visits
☐ Vaccines
☐ Routine cancer screenings
☐ Other preventive care (e.g., flu shots, dental visits, etc.)
☐ Other (please specify): ___________
☐ No, I have not had anything delayed or canceled.
☐ Don't know

30. How has the quality of your healthcare changed because of the coronavirus/COVID-19 pandemic?

☐ It's better
☐ It's the same
☐ It's worse

31. Have you had any telehealth visits that replaced an office visit because of the coronavirus/COVID-19 pandemic?

☐ Yes
☐ No

31a. (If yes to 31), how would you rate your overall experience with telehealth visits because of the coronavirus/COVID-19 pandemic?

☐ Satisfactory
☐ Undecided/neutral
☐ Unsatisfactory

32. **Please select responses below for the following support services that you may have needed or not needed, and received or not received <u>because of the coronavirus/COVID-19 pandemic</u>.**

	Needed, received	Needed, not received	Not Needed
Participate in a support group	☐	☐	☐
Have a paid caregiver or home health aide come to my house	☐	☐	☐
Have a nurse come to my house	☐	☐	☐
See a psychiatrist, psychologist or other mental health professional	☐	☐	☐
See a social worker	☐	☐	☐
See a pain management expert	☐	☐	☐
See a physical or occupational therapist	☐	☐	☐
See a palliative or supportive care expert	☐	☐	☐
Talk with a religious or spiritual counselor	☐	☐	☐
Other (please specify):	☐	☐	☐

Please answer the following questions if you were employed at the beginning of the <u>coronavirus/COVID-19 pandemic.</u>

33. **Has your employment status changed in any of the following ways, since March 2020 due to the COVID- 19 pandemic? Please select all that apply.**

- ☐ Decreased hours worked
- ☐ Increased hours worked
- ☐ Changed to work from home/telework
- ☐ Furloughed (i.e., a required suspension or decrease in hours on a temporary basis, an unpaid leave of absence)
- ☐ Laid off or lost employment
- ☐ Voluntarily left employment/quit
- ☐ Decided to retire early
- ☐ Returned to the work force
- ☐ No change in employment specifically due to the COVID-19 pandemic

Note: *Only answer 33a-c, if you **DID NOT** check* **returned to the work force, increased hours worked, or no change in employment:**

33a. Did any of the following reasons cause or lead to this change in your work status? Please select all that apply.

☐ My employer made the change
☐ I asked for a change in my status to reduce my risk of exposure to COVID-19 while at work
☐ I had problems physically getting to work, which forced a change in my work status
☐ I had to change my work status to care for someone with COVID-19
☐ I changed my work status because I had children out of school or needed to care for a relative or friend at home.

33b. Did the change in your employment status due to the COVID-19 pandemic impact your health insurance status?

☐ Yes
☐ No
☐ I am not sure

33c. What was the impact on your health insurance?

☐ I lost my health insurance and I still do not have coverage
☐ I lost my health insurance for a little while, but I now have coverage
☐ I lost my health insurance but was able to immediately have new coverage
☐ I am not sure

Note: *Only answer 33d, if you checked* **returned to the work force or increased hours worked:**

33d. Did any of the following reasons cause or lead to this change in employment status?

Please select all that apply.

☐ There was an increased demand for the type of work I do.
☐ Financial need
☐ Other ____

Thank you for answering these questions.
