# Supplementary material for: Lessening the Impact of Financial Toxicity (LIFT): a protocol for a multi-site, single-arm trial examining the effect of financial navigation on financial toxicity in adult patients with cancer in rural and non-rural settings
Source: Trials. 2022 Oct 3;23:839. doi: 10.1186/s13063-022-06745-4 (PMC9527389; doi:10.1186/s13063-022-06745-4)
Supplement: Supplementary file 4 — Additional file 4. Intake Form – includes the form used by navigators to assess patient financial needs and potential resources [file 13063_2022_6745_MOESM4_ESM.pdf]

Study ID: ___________ **Study Site**: ____________ **Date**: ____________ **FN**: ___________

FINANCIAL ASSISTANCE INTAKE APPLICANT INFORMATION

Using plain, clear language explain the application questions are to help better navigate complex cancer-related finances and to study if this kind of assistance approach is helpful. Inform patient that although we will be asking about specific expenses, bills, and debts, this is more to get an overall picture rather than suggest we will be able to help with each of these items. Tell pt. that everything is confidential. Let pt. know that if they do not know the answers to all of these questions, they will receive a list of items they need to bring in for the next appointment with you. Keep this physical document with answers recorded stored in a secure, locked location where no one but you has access for confidentiality.

**Name:**		**MRN#:**	
**Date of Birth:**		**County**:	
**Best Contact #:**		**Email:**	
**Baseline COST Date:**		**COST Score**:	

A. DEMOGRAPHICS

Are you:<br>☐ Male<br>☐ Female<br>☐ Non-binary<br>☐ I prefer not to answer<br><br>Are you:<br>☐Married<br>☐Divorced<br>☐Separated<br>☐Widowed<br>☐ Never married<br>☐ Unmarried but living with a partner<br>☐ I prefer not to answer	How would you describe your current employment situation? Please select what best describes your employment situation.<br><br>**CURRENTLY WORKING**<br>**How many FULL time (**35 hrs or more/wk) **jobs do you have?_________**<br><br>**How many PART time** (less than 35 hrs/wk) **jobs do you have?**<br>_________<br><br>**NOT CURRENTLY WORKING**<br>☐ On paid sick leave<br>☐ On unpaid sick leave<br>☐ On disability<br>☐ Retired<br>☐ Not employed- looking for a job<br>☐ Not employed- not looking for a job<br>☐ Not working-other: please describe___________________
Which of the following would you say is your race? Choose all that apply to you.<br>☐ White<br>☐ Black or African American<br>☐ Asian<br>☐ Native Hawaiian or Pacific Islander<br>☐ American Indian or Alaskan Native<br>☐ Other: please describe______________<br>☐ I prefer not to answer	What is the highest level of formal education you have completed?<br>☐ Less than high school<br>☐ High school graduate or GED<br>☐ Some college or technical or vocational school<br>☐ College graduate<br>☐ Some graduate or professional school<br>☐ Completed graduate or professional school<br>☐ I prefer not to answer
Are you of Hispanic, Latino/a or Spanish in origin?<br>☐ Yes ☐ No	Do you speak a language other than English at home?<br>☐ Yes<br>☐ No<br>☐ Don't know<br>☐ I prefer not to answer<br>IF YES<br>What is this language?<br>☐ Spanish<br>☐ Other, please specify: ____

Study ID: ___________ **Study Site**: ____________ **Date**: ____________ **FN**: ___________

Treatment Questions:

What was your primary cancer diagnosis?	What's your current cancer treatment status?
☐ Breast<br>☐ Colon or Rectal<br>☐ Lung<br>☐ Leukemia/Lymphoma<br>☐ Other: please describe _________________	☐ Cancer treatment plan in development<br>☐ In cancer treatment currently<br>☐ Done with cancer treatment<br>☐ Receiving supportive care or palliative care only
When did you receive your initial diagnosis?<br>Month__________ Year__________	Have you had a recurrence?<br>☐ Yes ☐ No<br>When did the recurrence occur? Month__________ Year_________

What kind of cancer treatment or services have you received since being diagnosed with cancer? *(Check all that apply)*

	Treatment plan in development	In treatment currently	Finished Treatment	Date complete
Surgery				
Chemotherapy				
Radiation therapy				
Immunotherapy				
Hormonal/Endocrine therapy				
Supportive or palliative care				
Other: please describe				

**B. FINANCIAL INFORMATION:**			**NOTES**
**1.**	What is your monthly income over the past 6 weeks?<br>Included all forms of income, for example, wages, retirement, Social Security, child support, Disability, food stamps	$	
**2.**	Has your income changed because of cancer?<br>If **yes** go to #2a if **no** go to # 3	☐Yes ☐ No	
	a. If yes, has it increased or decreased?<br>If decreased go to #2b	☐Increased<br>☐ Decreased	
	b. If decreased, why or in what ways has it decreased since having cancer?<br>If there is a decrease in income after cancer diagnosis, describe reason e.g. unpaid time-off for medical appointments, unable to perform physical work duties following surgery, laid-off/loss of job because of taking too much time off for medical treatment.		
	c. Have you or anyone in your home lost income due to the Covid-19 pandemic?	☐ Yes ☐ No	
	d. If yes, why or in what ways has it decreased since the Covid-19 pandemic started?<br>If there is a decrease in income after cancer diagnosis, describe reason e.g. unpaid time-off for medical appointments, unable to perform physical work duties following surgery, laid-off/loss of job because of taking too much time off for medical treatment.		
**3.**	Are you receiving income from Social Security or Disability SSI or SSDI?	☐Yes ☐ No<br>☐ Not Currently Eligible<br>☐ Document collection in process	
	a. If yes, what is the date you began receiving it?	Date:	

	b. If no, is there an application or appeal in process?	☐Yes ☐ No	
**4.**	Any **other** applications or appeals in process.	☐Yes ☐ No	
**5.**	If any **other** applications or appeals in process. Note application name below and status to the right.<br>Other Application or appeal: ______________________________________	☐ Approved<br>☐ Denial<br>☐ Still in process	
**6.**	How many people do you live with?<br>Make note of total # of individuals in household	#	
**7.**	Do you have any dependents?	☐Yes ☐ No	
	a. If yes, how many dependents are children?	#	
**8.**	Do any of the people who live in your household have a job?	☐Yes ☐ No	
	a. If yes, how many household members have a job?<br>b. Is it full time or part-time?	#<br>☐ Full-time<br>☐ Part-time	

**C. EXPENSES (BILLS/DEBT)**			**NOTES**
**9.**	Do you own or rent your home? Other? If Other (explain)<br>If other (i.e. staying with family or friend, roommates: temporary housing) please describe:	☐ Rent<br>☐ Own<br>☐ Other	
**10.**	About how much do you pay in monthly rent/mortgage?	$	
**11.**	About how much do you pay in monthly utilities?<br>Includes patient's share of electricity, gas, water, sewer, garbage, cell phone bill, if applicable	$	
**12.**	About how much do you pay for **other** monthly expenses, not including rent/mortgage and utilities?	$	
**13.**	Are you facing possible eviction if you rent, or home foreclosure if you own your home, due to loss of income during the COVID-19 pandemic?	☐Yes ☐ No	
**14.**	Do you have reliable transportation?	☐ Yes ☐ No	
	a. If yes, what are your monthly transportation costs?	$	
**15.**	Do you have credit card debt?	☐ Yes ☐ No	
	a. If yes, about what are your monthly credit card costs?	$	
**16.**	Do you have car loans?	☐ Yes ☐ No	
	a. If yes, about what are your monthly car loan costs?	$	
**17.**	Do you have insurance bills?	☐ Yes ☐ No	
	a. If yes, about what are your monthly insurance bill costs?	$	
**18.**	Do you have **other** debt?	☐ Yes ☐ No	
	a. If yes, about what are the monthly costs?	$	
**19.**	Please describe:		
	**Total household monthly expenditures:**	$	

**D. ASSETS/SAVINGS**			**NOTES**
**20.**	Do you have a Savings and/or Checking Account?	☐ Yes ☐ No	
	a. If yes, about how much money you have in your savings and checking account(s) combined?	$	
**21.**	Do you have retirement accounts?	☐ Yes ☐ No	
	a. If yes, about how much money do you have in your retirement account(s)?	$	

**22.**	Do you have investments accounts?	☐ Yes ☐ No	
	a. If yes, about how much money do you have in these account(s)?	$	
**23.**	Do you have 2 months of living expenses saved?	☐ Yes ☐ No	
**24.**	If you have a spouse, do they work outside the home for income?	☐ Yes ☐ No<br>☐ Not Applicable	
**25.**	If you have a spouse, does your spouse have a retirement account?	☐ Yes ☐ No<br>☐ Not Applicable	
	a. If yes, about how much money do you have in that account(s)?	$	
**26.**	If you have a spouse, does your spouse have an investment account?	☐ Yes ☐ No<br>☐ Not Applicable	
	a. If yes, about how much money do you have in that account(s)?	$	
**Comments:** Add any other pt. comments about financial status, i.e. amount of items above?			

**E. EMPLOYER BASED BENEFITS/INCOME**			**NOTES**
**27.**	**Medical Leave of Absence:** Are you on a MLOA of absence from your job?	☐ Yes ☐ No	
	a. If **yes**, is your employer offering you FMLA?<br>**Do you know what FMLA is?**<br>An employer that has more than 50 employees must offer 12 weeks of leave where your job is preserved for 12 weeks. This means your job can't be taken away, but it does not mean you are guaranteed to be paid during this time.<br>**Do you know the difference between continuous vs. intermittent Family Medical Leave?** If **no**, explain: Continuous FMLA leave employee is absent for more than three consecutive business days and has been treated by a doctor. Intermittent FMLA leave is when an employee is taking time off in separate blocks due to a health condition that qualifies for FMLA.	☐ Yes ☐ No	
**28.**	**Short term and long-term disability insurance:** Do you receive short-term employer-based disability insurance through your employer?	☐ Yes ☐ No<br>☐ Don't Know	
	a. If **yes**, will your employer-based health insurance continue during short-term disability?	☐ Yes ☐ No<br>☐ Don't Know	

**F. HEALTH INSURANCE & COVERAGE**			**NOTES**
**29.**	Do you have health insurance?<br>If **yes**, go to 30 If **no**, go to 29	☐ Yes ☐ No	
**30.**	Have you lost health insurance coverage due to the COVID-19 pandemic?<br>**Regardless of answer go to Section K**	☐ Yes ☐ No	
**31.**	If yes, what kind?<br>☐ Employer based or Student Health Insurance: **skip to Section G**<br>☐ Private Health Insurance (including Affordable Care Act) **skip to Section H**<br>☐ Medicare: **skip to Section I**<br>☐ Medicaid: **skip to Section J**<br>☐ Military health care (TRICARE, VA, CHAMP-VA)<br>☐ Other: ______________________________		

**G. EMPLOYER BASED OR STUDENT HEALTH INSURANCE**			**NOTES**
**32.**	What is the source of your insurance?<br>Important to ask if employer based to see if they can get COBRA	☐ Employer<br>☐ Student	
**33.**	**Employer Based Coverage:** Does the employer-based health insurance cover the patient only?	☐ Yes ☐ No	
	a. If no, is it family/dependent coverage as well?	☐ Yes ☐ No	

**34.**	Who pays the health insurance premium during employment?	☐ Employer<br>☐ Patient	
**35.**	How much is the monthly premium cost?	$	
**36.**	Do you understand your annual deductible, co-payment/coinsurance, and annual out-of pocket maximums?<br>b. If no, refer to financial counselor for clarification		
**37.**	**Risk of Loss of Employment:** Do you feel there is a risk that you might lose your employment and lose employer-based health insurance?<br>a. If yes, see Section K: Eligible for Cobra Care?	☐ Yes ☐ No	
**H. PRIVATE/ACA HEALTH INSURANCE**			**NOTES**
**38.**	Do you receive federal tax subsidies to cover a portion or all of the cost of the health insurance premiums?	☐ Yes ☐ No	
**39.**	Do you know what your health insurance premiums are?<br>a. If yes, what are they?	☐ Yes ☐ No<br>$	
**40.**	Do you understand your annual deductible, co-payment/coinsurance, and annual out-of pocket maximums?	☐ Yes ☐ No	
**41.**	Is there a drug/pharmacy benefit?	☐ Yes ☐ No	
**42.**	Will you be able to continue the private plan if there is a disruption in income?	☐ Yes ☐ No	
**I. MEDICARE**			**NOTES**
**43.**	Do you have Medicare Parts A and B?	☐ Yes ☐ No	
**44.**	Do you have Medicare Prescription Drug Coverage (Part D)?	☐ Yes ☐ No	
**45.**	Do you have Medicare Advantage or Medicap?	☐ Yes ☐ No	
**46.**	Extra Help (i.e., when DSS pays Part D premium)?<br>a. If eligible but not covered, why? ___________________________	☐ Yes ☐ No	
**J. MEDICAID**			**NOTES**
**47.**	Do you have Full Medicaid?<br>Full Medicaid covers all medical and pharmacy costs	☐ Yes ☐ No	
	a. If **no**, do you have MQB Medicaid (Part D premium payment)?	☐ Yes ☐ No	
	b. If **no**, do you have Family planning Medicaid?	☐ Yes ☐ No	
	c. If **no**, do you have a MEDICAID application in process?	☐ Yes<br>☐ Not currently eligible<br>☐ Documents collection in progress	
**48.**	Do you have a deductible with Medicaid?	☐ Yes ☐ No	
**K. UNINSURED**			**REFERRAL?**
**49. Eligible for ACA?**<br>• To be eligible for health insurance and a subsidy through the ACA you must have income between 100% and 400% of the Federal Poverty Limits (FPL). For a single adult that's between $12,000 and $47,000 annual income. Open Enrollment Is in November of each year. https://obamacarefornc.com/subsidies/ | https://www.healthcare.gov/<br>• Special Enrollment: you may enroll outside of the open enrollment period if you experience a qualifying event, such as loss of health insurance. You must enroll within 60 days of the qualifying event.			☐ Yes ☐ No
**50. Eligible for Financial Assistance (charity care)?**<br>The financial assistance program for medical bills is for low income patients.			☐ Yes ☐ No

**51. Eligible for Cobra Care?**<br>**COBRA Care** may assist with payment of COBRA insurance premium payments for 18 months while an active patient. Discuss COBRA Care (Discuss rules regarding COBRA, continuation of coverage for 18 months, need to complete election of coverage paper work)	☐ Yes ☐ No
**52. Eligible for Medicaid?**<br>Medicaid Eligibility: Federally funded health insurance administered by your county Department of Social Services. Disabled adults and elderly with income below 100% of the federal poverty limit may be eligible for Medicaid. Most adults must be determined disabled by the Social Security Disability Administration, uninsured and low income to qualify.	☐ Yes ☐ No
**53. Eligible or need help with Medicare?**<br>Refer to Seniors Health Insurance Information Program SHIIP for help with selecting Medicare plans and determining eligibility for extra help. https://www.ncdoi.com/SHIIP/	☐ Yes ☐ No
**54. Eligible for Social Security Disability Insurance (SSDI)?**<br>Social Security https://www.ssa.gov/benefits/disability/ pays benefits to people who can't work because they have a medical condition that's expected to last one year or more.<br>• **SSI**: Supplemental Security Income is for individuals who are determined to be disabled by the Social Security Disability Service and are very low income and do not have a work history.<br>• **SSDI**: Social Security Disability Insurance is for individuals who are determined to be disabled by the Social Security Disability Service and have met the earnings requirement. The benefit is based on your contribution.	☐ Yes ☐ No

Thank you for taking this time. We'll move on to seeing what sources of financial assistance we can find for you!

INSTRUCTIONS FOR FILLING OUT INITIAL APPOINTMENT SUMMARY:

1) **Next Appointment**:
   a. Verify date, time and location or if by phone
   b. Verify contact information
2) **Possible Eligibility or Referrals list:**
   a. Fill in blank slots with possible resources or referrals the participants is eligible for. You can use the checklist below.

- ☐ Assistance from Private Charitable Foundations
- ☐ Assistance with the cost of Medicare Part D (Extra Help)
- ☐ Chaplain
- ☐ Counseling
- ☐ Credit Counseling
- ☐ Employer- based disability Insurance
- ☐ Social Security Disability SSI or SSDI
- ☐ Housing/ Lodging Assistance
- ☐ Legal Clinic Referral
- ☐ Health Insurance Subsidy from ACA
- ☐ Medicaid
- ☐ Medicare Enrollment Assistance (SHIIP)
- ☐ Oncology Out-Patient Social Work Referral
- ☐ Pharmaceutical Manufacturer Assistance Program (MAP)
- ☐ Transportation Assistance
- ☐ Charity Care
- ☐ Cobra Care
- ☐ Veterans Services
- ☐ Other: Please describe__________________
- ☐ Local/Site Specific Program: Please describe :____________

3) **Documents Needed for Next Appointment**
   a. Review list of documents that patient will need for next visit.
