# Supplementary material for: Lessening the Impact of Financial Toxicity (LIFT): a protocol for a multi-site, single-arm trial examining the effect of financial navigation on financial toxicity in adult patients with cancer in rural and non-rural settings
Source: Trials. 2022 Oct 3;23:839. doi: 10.1186/s13063-022-06745-4 (PMC9527389; doi:10.1186/s13063-022-06745-4)
Supplement: Supplementary file 5 — Additional file 5. Check-in Form – includes the form used by navigators to follow-up on patient financial needs and receipt of resources throughout the study [file 13063_2022_6745_MOESM5_ESM.pdf]

Study ID: ___________ **Study Site:** ____________ **Date:** ____________ **FN:** ___________

Financial Assistance Check-In Form

A. CONTACT

Please review participant's information including which referrals and benefits were suggested for the patient and complete Section A before contacting participant

Initial Assessment Date:

**Type of Contact:**	**If Unable to Contact:**
☐No Contact Needed<br>☐Unable to Contact<br>☐Phone<br>☐In person at clinic<br>☐In person elsewhere (please describe): _________________	☐ No answer<br>☐ Phone disabled<br>☐ Lost touch<br>☐ Other: please describe

GROUP 1 CHECK IN SCRIPT:

Hello, Mr./Ms./. [XXXX]. My name is [XXXX] and I'm calling from [XXXX]. I spoke to you on [enter date] about helping you apply for some financial help. Last time we talked, we reviewed your financial situation and checked into any financial assistance that you might be eligible for. We talked about a few options and I wanted to follow up on that conversation.

Is now a good time to chat? (if no, schedule another time-see line below. If yes, continue)

So let's review where we are in the process and what I can do to help. A lot of people have questions about this information. What sort of questions do you have?

Rescheduled Date, if needed:	

B. PREVIOUS LIFE STATUS

	Last time we spoke your status was [**LOOK IN CHECK IN FORM SUMMARY**]	**Has it Changed?**	**Current Status?**
Marital Status		☐Yes<br>☐No	☐Married<br>☐Divorced<br>☐Separated<br>☐Widowed<br>☐ Never married<br>☐ Unmarried but living with a partner<br>☐ I prefer not to answer
Employment		☐Yes<br>☐No	☐ How many FULL time (35 hrs or more/wk) jobs do you have?_____<br>☐ How many PART time (less than 35 hrs/wk) jobs do you have? ________<br>☐ On paid sick leave<br>☐ On unpaid sick leave<br>☐ On disability<br>☐ Retired<br>☐ Not employed- looking for a job<br>☐ Not employed- not looking for a job<br>☐ Not working-other
Monthly Income		☐Yes<br>☐No	$
Insurance		☐Yes<br>☐No	Do you have health insurance?<br>☐Yes<br>☐No
Housing		☐Yes<br>☐No	Do you own or rent your home? Other? If Other (explain)<br>☐ Rent<br>☐ Own<br>☐ Other:_______________

Study ID: ___________ **Study Site:** ____________ **Date:** ____________ **FN:** ___________

C. REFERRALS

Review individual list of referrals given to participant (**from Initial Appointment Summary below**) and answer for each referral as completely as possible at this time. If you have a new referral to give during this check-in, please write date/info below.

You can informally ask: Did you get in touch with [NAME OF REFERRAL]? If yes, can you tell me: What's the plan? Were there any decisions? If you have new information or suggestions say: I thought it would be a good idea for you to try [XXXX] organization. Here is the name and contact information. Let me know if you have any questions or need any help.

Possible Referrals: [LOOK IN INITIAL APPOINTMENT SUMMARY]

Counseling:	☐Yes ☐No	Veteran Services	☐Yes ☐No
Legal Clinic Referral	☐Yes ☐No	Chaplain	☐Yes ☐No
Oncology Out-Patient Social Work Referral	☐Yes ☐No	Credit Counseling:	☐Yes ☐No

If new referral given at this check-in write date/info below

	Contact made?	Barriers to contacting?	Date process (if available)		Follow-up needed?/Notes
			Started	Ended	
Counseling	☐Yes ☐No ☐In progress	Hospitalized ☐ Other: describe _______________			
NC Legal Clinic Referral	☐Yes ☐No ☐In progress	☐Hospitalized ☐ Other: describe ______________			
Oncology Out-Patient Social Work Referral	☐Yes ☐No ☐In progress	☐Hospitalized ☐ Other: describe ______________			
Veterans Services	☐Yes ☐No ☐In progress	☐Hospitalized ☐ Other: describe ______________			
Chaplain	☐Yes ☐No ☐In progress	☐Hospitalized ☐ Other: describe ______________			
Credit Counseling	☐Yes ☐No ☐In progress	☐Hospitalized ☐ Other: describe ______________			

D. BENEFITS

Review individual list of possible benefit eligibility for participant (from Post First Visit Checklist below) and answer for each benefit as completely as possible at this time. If you have a new benefit to add during this check-in , please write date/info below.
You can informally ask: Did you get in touch with XXXXXX? If yes, can you tell me: What's the plan? Were there any decisions? If at first visit there was not specific benefit information given, but you have new information or suggestions say: I thought it would be a good idea for you to try X organization. Here is the name and contact information. Let me know if you have any questions or need any help.

Possible Benefits: [LOOK IN INITIAL APPOINTMENT SUMMARY]

Assistance from Private Charitable Foundations	☐Yes ☐No	Pharmaceutical Manufacturer Assistance Program (MAP)	☐Yes ☐No
Assistance with the cost of Medicare Part D (Extra Help)	☐Yes ☐No	Transportation Assistance	☐Yes ☐No
Health Insurance ACA Subsidy	☐Yes ☐No	Hospital-based Financial Assistance (Charity Care)	☐Yes ☐No
Medicaid	☐Yes ☐No	Site Cobra Care	☐Yes ☐No
Employer- based Disability	☐Yes ☐No	Local/Site Specific Program	☐Yes ☐No

Study ID: ___________ **Study Site**: ____________ **Date**: ____________ **FN**: ___________

Social Security Disability SSI or SSDI	☐Yes ☐No	Local/Site Specific Program	☐Yes ☐No
Housing/Lodging Assistance	☐Yes ☐No	Other Benefit 1	☐Yes ☐No
Medicare Enrollment Assistance (SHIIP)		Other Benefit 2	☐Yes ☐No
**If new referral given at this check-in write date/info below**			

	Application	Status	If denial, appeal in process?	Barriers to filing?	Date process If available)		Benefit Amt?	Follow-up needed?
					Started	Ended		
Private Charitable Foundations list: ______________________ ______________________	☐Yes<br>☐ Not currently eligible<br>☐ Document collection in progress	☐Approved<br>☐Denied<br>☐Still in process	☐Yes<br>☐No	☐ Hospitalized<br>☐Other: describe<br>______________<br>______________				☐Yes<br>☐No
Assistance with the cost of Medicare Part D (Extra Help)	☐Yes<br>☐ Not currently eligible<br>☐ Document collection in progress	☐Approved<br>☐Denied<br>☐Still in process	☐Yes<br>☐No	☐ Hospitalized<br>☐Other: describe<br>______________<br>______________				☐Yes<br>☐No
Health Insurance ACA Subsidy	☐Yes<br>☐ Not currently eligible<br>☐ Document collection in progress	☐Approved<br>☐Denied<br>☐Still in process	☐Yes<br>☐No	☐ Hospitalized<br>☐Other: describe<br>______________<br>______________				☐Yes<br>☐No
Medicaid	☐Yes<br>☐ Not currently eligible<br>☐ Document collection in progress	☐Approved<br>☐Denied<br>☐Still in process	☐Yes<br>☐No	☐ Hospitalized<br>☐Other: describe<br>______________<br>______________				☐Yes<br>☐No
Employer- based Disability	☐Yes<br>☐ Not currently eligible<br>☐ Document collection in progress	☐Approved<br>☐Denied<br>☐Still in process	☐Yes<br>☐No	☐ Hospitalized<br>☐Other: describe<br>______________<br>__________				☐Yes<br>☐No
Social Security Disability SSI or SSDI								
Housing/Lodging Assistance	☐Yes<br>☐ Not currently eligible<br>☐ Document collection in progress	☐Approved<br>☐Denied<br>☐Still in process	☐Yes<br>☐No	☐Hospitalized<br>☐Other: describe<br>________________<br>____________				☐Yes<br>☐No
Medicare Enrollment Assistance (SHIIP)	☐Yes<br>☐ Not currently eligible<br>☐ Document collection in progress	☐Approved<br>☐Denied<br>☐Still in process	☐Yes<br>☐No	☐Hospitalized<br>☐ Other: describe<br>_______________<br>_________				☐Yes<br>☐No
Pharmaceutical Manufacturer Assistance Program (MAP)	☐Yes<br>☐ Not currently eligible<br>☐ Document collection in progress	☐Approved<br>☐Denied<br>☐Still in process	☐Yes<br>☐No	☐Hospitalized<br>☐ Other: describe<br>______________<br>__________				☐Yes<br>☐No

Study ID: ___________ **Study Site**: ____________ **Date**: ____________ **FN**: ___________

Transportation Assistance	☐Yes<br>☐ Not currently eligible<br>☐ Document collection in progress	☐Approved<br>☐Denied<br>☐Still in process	☐Yes<br>☐No	☐Hospitalized<br>☐ Other: describe<br>______________<br>__________				☐Yes<br>☐No
Hospital-based Financial Assistance (Charity Care)	☐Yes<br>☐ Not currently eligible<br>☐ Document collection in progress	☐Approved<br>☐Denied<br>☐Still in process	☐Yes<br>☐No	☐Hospitalized<br>☐ Other: describe<br>______________<br>__________				☐Yes<br>☐No
Site Cobra Care	☐Yes<br>☐ Not currently eligible<br>☐ Document collection in progress	☐Approved<br>☐Denied<br>☐Still in process	☐Yes<br>☐No	☐Hospitalized<br>☐ Other: describe<br>______________<br>__________				☐Yes<br>☐No
Other Benefit 1	☐Yes<br>☐ Not currently eligible<br>☐ Document collection in progress	☐Approved<br>☐Denied<br>☐Still in process	☐Yes<br>☐No	☐Hospitalized<br>☐ Other: describe<br>______________<br>__________				☐Yes<br>☐No
Other Benefit 2	☐Yes<br>☐ Not currently eligible<br>☐ Document collection in progress	☐Approved<br>☐Denied<br>☐Still in process	☐Yes<br>☐No	☐Hospitalized<br>☐ Other: describe<br>______________<br>__________				☐Yes<br>☐No
Other Benefit 3	☐Yes<br>☐ Not currently eligible<br>☐ Document collection in progress	☐Approved<br>☐Denied<br>☐Still in process	☐Yes<br>☐No	☐Hospitalized<br>☐ Other: describe<br>______________<br>__________				☐Yes<br>☐No
**Notes:**								
**Local/Site Specific Program**								
Local/Site Specific Program	☐Yes<br>☐ Not currently eligible<br>☐ Document collection in progress	☐Approved<br>☐Denied<br>☐Still in process	☐Yes<br>☐No	☐Hospitalized<br>Other: describe<br>______________<br>__________				☐Yes<br>☐No
Local/Site Specific Program	☐Yes<br>☐ Not currently eligible<br>☐ Document collection in progress	☐Approved<br>☐Denied<br>☐Still in process	☐Yes<br>☐No	☐Hospitalized<br>☐ Other: describe<br>______________<br>__________				☐Yes<br>☐No
Local/Site Specific Program	☐Yes<br>☐ Not currently eligible<br>☐ Document collection in progress	☐Approved<br>☐Denied<br>☐Still in process	☐Yes<br>☐No	☐Hospitalized<br>☐ Other: describe<br>______________<br>__________				☐Yes<br>☐No
**Notes:**								

Next Check-in date:

Check schedule and next appointment date. Confirm if date still works. If it does not, reschedule.	
**Next Appt Date:**	
**Date:**	
**Location:**	

Study ID: ___________ **Study Site**: ____________ **Date**: ____________ **FN**: ___________

**Notes:**

**PAF REFERRAL**	
**Date:**	
**PAF Case Manager:**	
**Assistance with:**	
**Notes:**	
