# Supplementary material for: Lessening the Impact of Financial Toxicity (LIFT): a protocol for a multi-site, single-arm trial examining the effect of financial navigation on financial toxicity in adult patients with cancer in rural and non-rural settings
Source: Trials. 2022 Oct 3;23:839. doi: 10.1186/s13063-022-06745-4 (PMC9527389; doi:10.1186/s13063-022-06745-4)
Supplement: Supplementary file 6 — Additional file 6. Follow-up Survey – includes the patient experience survey given to patients following the intervention and a patient perspective survey about the intervention [file 13063_2022_6745_MOESM6_ESM.pdf]

Study ID: ___________ **Study Site**: ____________ **Date**: ____________ **FN**: ___________

Post Patient Experience Questionnaire

Please answer the following questions so that we can better understand your experience as a patient with cancer. Your answers to these questions will help us learn what concerns you most about your financial situation and ability to get healthcare. Please feel free to ask (your LIFT financial navigator) any questions you have about this survey.

THIS SET OF QUESTIONS ASKS ABOUT YOUR FINANCIAL SITUATION <u>SINCE BEING DIAGNOSED WITH CANCER</u>:

1. **Over the past few months have you stayed in a job because you were concerned about losing your health insurance?**

   ☐ Yes
   ☐ No
   ☐ N/A (e.g., have not had a job since diagnosis)

2. **Since your diagnosis, what out-of-pocket costs (i.e., costs to you that insurance doesn't cover) have you or your family had related to your health (Please mark all that apply).**

   ☐ Medications (e.g., prescription drugs and over-the-counter medications)
   Note: *Only answer 2a, if you checked Medications*
   2a. Was this cost expected or unexpected?
   ☐ Expected
   ☐ Unexpected

   ☐ Home healthcare (e.g., nursing, home medical supplies)
   Note: *Only answer 2b, if you checked Home healthcare*
   2b. Was this cost expected or unexpected?
   ☐ Expected
   ☐ Unexpected

   ☐ Counseling services (e.g., therapy, grief counseling)
   Note: *Only answer 2c, if you checked Counseling services*
   2c. Was this cost expected or unexpected?
   ☐ Expected
   ☐ Unexpected

   ☐ Rehabilitation therapies (e.g., occupational, speech, or physical therapy)
   Note: *Only answer 2d, if you checked Rehabilitation therapies*
   2d. Was this cost expected or unexpected?
   ☐ Expected
   ☐ Unexpected

☐ Alternative therapies (e.g., acupuncture, massage, hypnosis)
Note: *Only answer 2e, if you checked Alternative Therapies*
2e. Was this cost expected or unexpected?
☐ Expected
☐ Unexpected

☐ Other healthcare costs (e.g., copays for office or ER visits, additional referrals, imaging and lab tests)
Note: *Only answer 2f, if you checked Other healthcare costs*
2f. Was this cost expected or unexpected?
☐ Expected
☐ Unexpected

☐ Lodging associated with your healthcare (e.g., hotel costs)
Note: *Only answer 2g, if you checked Lodging*
2g. Was this cost expected or unexpected?
☐ Expected
☐ Unexpected

☐ Transportation associated with your healthcare (e.g., parking, gas)
Note: *Only answer 2h, if you checked Transportation*
2h. Was this cost expected or unexpected?
☐ Expected
☐ Unexpected

☐ Diet (e.g., enteral feeds, formula)
Note: *Only answer 2i, if you checked Diet*
2i. Was this cost expected or unexpected?
☐ Expected
☐ Unexpected

☐ I have not had any out of pocket costs related to my healthcare.

☐ Other:____

Note: *Only answer 2j, if you checked Other*
2j. Was this cost expected or unexpected?
☐ Expected
☐ Unexpected

3. Have you <u>ever</u> stopped or refused a treatment related to your cancer because it cost too much?
☐ Yes
☐ No

4. Have you <u>ever</u> been contacted by a debt collection agency as a result of your cancer care?

☐ Yes

☐ No

5. Which of the following have you <u>ever</u> done to manage the cost of your cancer care? Select all that apply

Skipped a vacation or other activity due to cost	☐
Borrowed money from friends, family, a bank, or other source	☐
Taken money from a savings or retirement account	☐
Filed for bankruptcy	☐
Avoided treatment for another medical problem (other than your cancer)	☐
Skipped paying non-medical bills like rent, credit cards, or other necessities	☐
Reduced spending on basics like food or clothing	☐
Sold stocks or investments	☐
Mortgaged, refinanced, or sold home	☐
Moved or downsized your home	☐

6. <u>Within the past few months</u>, how often did you do the following due to cost?

	**Never**	**Rarely**	**Sometimes**	**Often**
Delayed filling your prescribed medication	☐	☐	☐	☐
Filled only part of your prescribed medication	☐	☐	☐	☐
Avoided making your clinic visits	☐	☐	☐	☐
Decided not to use medically-related services such as physical therapy	☐	☐	☐	☐
Decided not to have a medical test performed	☐	☐	☐	☐
Decided not to receive other non-cancer preventive care (such as flu shots, cancer screenings, dental cleanings)	☐	☐	☐	☐

7. <u>Within the past few months,</u> how often did you <u>not</u> see a medical provider because of transportation issues?

- o Never
- o Rarely
- o Sometimes
- o Often

8. <u>Within the past few months,</u> how often have you been worried about the cost of travel (e.g., miles, gas) or lodging (e.g., hotel, apartment) related to your cancer care?

- o Never
- o Rarely
- o Sometimes
- o Often

9. **<u>Within the past few months,</u> how much have you worried about how the cost of your cancer care has impacted your loved ones?**
   - Not at all
   - Some/a little
   - A lot
   - All the time/constantly

10. **<u>Within the past few months,</u> how often have you worried whether your food would run out before you got money to buy more?**
    - Often True
    - Sometimes True
    - Never True
    - Don't Know/Refused

11. **<u>Within the past few months,</u> how often did the food you bought just not last and you didn't have money to get more?**
    - Often True
    - Sometimes True
    - Never True
    - Don't Know/Refused

THE NEXT SET OF QUESTIONS ASKS ABOUT YOUR HOUSEHOLD EMPLOYMENT AND INCOME:

12. **Which best describes your job(s)?**

    If you have more than one job, please check all that apply. If you are not working, please tell us about the last job you had.
    - **Sales, technical, or administrative support** (for example, medical assistant, real estate agent, store manager, administrative assistant, sales associate)
    - **Service provider** (for example housekeeper, home attendant, police officer, food server, janitor, hair-dresser, aesthetician)
    - **Operator, fabricator, or laborer** (for example utility worker, driver, seamstress/tailor, factory worker, laundry worker)
    - **Managerial or professional specialty** (for example, accountant, computer specialist, attorney, teacher, health practitioner)
    - **Arts, media or athletics** (for example artist, entertainer, journalist)
    - **Other (please specify):** ________________________

13. Which would you say best describes your workplace(s)?

If you have more than one job, please *check all that apply*. If you are not working, please tell us about the last job you had.

- ○ **Service** (for example restaurant, hotel, housekeeping, homecare provider, tailor, laundry)
- ○ **Retail** (for example a shop or store)
- ○ **Government** (for example post-office, courts, police or fire department)
- ○ **Manufacturing** (for example factory worker)
- ○ **Finance, insurance, or real estate**
- ○ **Wholesale Trade**
- ○ **Transportation and public utilities**
- ○ **Construction**
- ○ **Healthcare/Medical**
- ○ **Legal**
- ○ **Education**
- ○ **Arts and Entertainment**
- ○ **Other** (please specify): __________

**14.**	Has your cancer diagnosis led to any of the following changes in your employment? Please mark all that apply.	☐ Retired early<br>☐ Left my job<br>☐ Decreased work hours<br>☐ Increased work hours<br>☐ Briefly changed work schedule to attend medical appointments<br>☐ Changed job or careers<br>☐ No change to employment<br>☐ Other, please specify:______
**15.**	Did you miss any days of work because of your cancer diagnosis?	☐ Yes<br>☐ No → *skip to #17*
**16.**	Did you use any of the following for absences from work due to your cancer diagnosis? Please mark all that apply.	☐ Family Medical Leave Act (FMLA)?<br>☐ Paid sick leave<br>☐ Paid vacation leave<br>☐ Unpaid leave<br>☐ None<br>☐ Other, please specify:______
**17.**	Did your doctor or a member of the healthcare team ever have a conversation with you about the impact that cancer may have on your ability to stay employed?	☐ Yes<br>☐ No<br>☐ Not sure

**18.**	Did you speak to your employer or Human Resources (HR) department about how cancer may impact your work ability to do your job?	☐ Yes<br>☐ No<br>☐ Not sure
**19.**	Are there ways that your employer supported you after you were diagnosed with cancer? Please mark all that apply.	☐ Changed job duties/responsibilities<br>☐ Allowed additional paid time off<br>☐ Offered assistance that helped cover medical and non-medical bills<br>☐ Offered assistance in understanding insurance benefits and coverage<br>☐ Discussed different leave options<br>☐ Provided option to work from home<br>☐ Provided flexible work schedule<br>☐ Other (please specify:_____)
**20.**	Are there ways that your employer could have better helped you since you were diagnosed with cancer? Please mark all that apply.	☐ Changed in job duties/responsibilities<br>☐ Allowed additional paid time off<br>☐ Offered assistance that helped cover medical and non-medical bills<br>☐ Offered assistance in understanding insurance benefits and coverage<br>☐ Discussed different leave options<br>☐ Provided option to work at home<br>☐ Provided flexible work schedule<br>☐ Other (please specify:_____)

THE NEXT SET OF QUESTIONS ASKS ABOUT YOUR GENERAL FINANCES:

21.How confident are you that you could find the money to pay for a *financial emergency* that costs about $1000?

1	2	3	4	5	6	7	8	9	10

No Confidence | Little Confidence | Some Confidence | High Confidence

22. How often do you find yourself "just getting by" financially or living *paycheck to paycheck?*

1	2	3	4	5	6	7	8	9	10

Never | Sometimes | Often | Always

23. How hard is it for you to pay for the very basics like food, housing, medical care, and heating?

1	2	3	4	5	6	7	8	9	10

Never | Sometimes | Often | Always

THIS FINAL SET OF QUESTIONS ASKS ABOUT HOW THE NOVEL CORONAVIRUS DISEASE (ALSO KNOWN AS COVID-19) HAS AFFECTED YOUR CANCER CARE AND FINANCES:

24. Has there ever been a time you had to delay or stop your cancer treatment because of the coronavirus/COVID-19 pandemic?

- ☐ Yes
- ☐ No

25. Have you had any of the following healthcare services delayed or canceled because of the coronavirus/COVID-19 pandemic?

- ☐ Surveillance monitoring (cancer monitoring)
- ☐ Lab tests
- ☐ Scans/imaging
- ☐ Cancer-related surgery
- ☐ Chemotherapy or other cancer-related infusion appointments
- ☐ Radiation therapy appointments
- ☐ Cancer-related follow-up visits
- ☐ Vaccines
- ☐ Routine cancer screenings
- ☐ Other preventive care (e.g., flu shots, dental visits, etc.)
- ☐ Other (please specify): ___________
- ☐ No, I have not had anything delayed or canceled.
- ☐ Don't know

26. How has the quality of your healthcare changed because of the coronavirus/COVID-19 pandemic?

☐ It's better

☐ It's the same

☐ It's worse

27. Have you had any telehealth visits, that replaced an office visit, because of the coronavirus/COVID-19 pandemic?

☐ Yes

☐ No

27a. (If yes to 27), how would you rate your overall experience with telehealth visits because of the coronavirus/COVID-19 pandemic?

☐ Satisfactory

☐ Undecided/neutral

☐ Unsatisfactory

28. Please select responses below for the following support services that you may have needed or not needed, and received or not received <u>because of the coronavirus/COVID-19 pandemic</u>.

	Needed, received	Needed, not received	Not Needed
**Participate in a support group**	☐	☐	☐
**Have a paid caregiver or home health aide come to my house**	☐	☐	☐
**Have a nurse come to my house**	☐	☐	☐
**See a psychiatrist, psychologist or other mental health professional**	☐	☐	☐
**See a social worker**	☐	☐	☐
**See a pain management expert**	☐	☐	☐
**See a physical or occupational therapist**	☐	☐	☐
**See a palliative or supportive care expert**	☐	☐	☐
**Talk with a religious or spiritual counselor**	☐	☐	☐
**Other (please specify):**	☐	☐	☐

Please answer the following questions if you were employed at the beginning of the coronavirus/COVID-19 pandemic.

29. Has your employment status changed in any of the following ways, since March 2020 due to the COVID- 19 pandemic? Please select all that apply.

- ☐ Decreased hours worked
- ☐ Increased hours worked
- ☐ Changed to work from home/telework
- ☐ Furloughed (i.e., a required suspension or decrease in hours on a temporary basis, an unpaid leave of absence)
- ☐ Laid off or lost employment
- ☐ Voluntarily left employment/quit
- ☐ Decided to retire early
- ☐ Returned to the work force
- ☐ No change in employment specifically due to the COVID-19 pandemic

Note: *Only answer 25a-c, if you* ***DID NOT*** *check* **returned to the work force, increased hours worked, or no change in employment:**

29a. Did any of the following reasons cause or lead to this change in employment status? Please select all that apply.

- ☐ My employer made the change
- ☐ I asked for a change in my status to reduce my risk of exposure to COVID-19 while at work
- ☐ I had problems physically getting to work, which forced a change in my work status
- ☐ I had to change my work status to care for someone with COVID-19
- ☐ I changed my work status because I had children out of school or needed to care for a relative or friend at home.

29b. Did the change in your employment status due to the COVID-19 pandemic impact your health insurance status?

- ☐ Yes
- ☐ No
- ☐ I am not sure

29c. What was the impact on your health insurance?

- ☐ I lost my health insurance and I still do not have coverage
- ☐ I lost my health insurance for a little while, but I now have coverage
- ☐ I lost my health insurance but was able to immediately have new coverage
- ☐ I am not sure

Note: *Only answer 29d, if you checked* **returned to the work force or increased hours worked:**

29d. Did any of the following reasons cause or lead to this change in employment status?

Please select all that apply.

☐ There was a increased demand for the type of work I do.

☐ Financial need

☐ Other ____

Thank you for answering these questions!

Study ID: ___________ **Study Site**: ____________ **Date**: ____________ **FN**: ___________

Patient Perspective Survey

Instructions: Please complete the following survey to let us know how you feel about the support you received from the LIFT financial navigation program.

	Strongly Disagree	Disagree	Neither Agree Nor Disagree	Agree	Strongly Agree
**1.** Overall, I was satisfied with the LIFT financial navigation help I received	1	2	3	4	5
**2.** I feel that participating in the LIFT financial navigation program made it easier for me to understand what financial help is available to me	1	2	3	4	5
**3.** I feel that participating in the LIFT financial navigation program made it easier for me to understand how to apply for financial assistance	1	2	3	4	5
**4.** I have fewer financial worries after participating in the LIFT financial navigation program	1	2	3	4	5
**5.** The LIFT financial navigation print materials I received were helpful	1	2	3	4	5
**6.** The LIFT financial navigation website was easy to use	1	2	3	4	5
**7.** Scheduling my financial navigation appointments was convenient	1	2	3	4	5
**8.** I wish there had been more financial navigation appointments	1	2	3	4	5
**9.** I felt comfortable talking with my financial navigator about my financial concerns	1	2	3	4	5
**10.** The financial navigation program addressed issues that were important to me	1	2	3	4	5
**11.** The financial navigation program was a good match for me and my life	1	2	3	4	5

We would like to get your feedback on your experience in this research study.
Directions: Please mark one response for each of the following questions.

12. Was it worthwhile for you to participate in the LIFT financial navigation program?

- ○ Yes
- ○ No
- ○ Uncertain

13. If you had to do it over, would you participate in the LIFT financial navigation program again?
- o Yes
- o No
- o Uncertain

14. Would you recommend participating in the LIFT financial navigation program to others?
- o Yes
- o No
- o Uncertain

15. Overall, did your quality of life change by participating in the LIFT financial navigation program?
- o It improved
- o It stayed the same
- o It got worse

16. Overall, how was your experience participating in the LIFT financial navigation program?
- o Better than I expected
- o The same as I expected
- o Worse than I expected

17. Please share any feedback you have about the financial navigation program as a whole. This could be your likes, dislikes, or any other general feedback:

18. If there was ONE thing you could change about the financial navigation program, what would it be?

This concludes our survey.
Thank you for taking the time to participate in our financial navigation program.
