# Supplementary material for: Lessening the Impact of Financial Toxicity (LIFT): a protocol for a multi-site, single-arm trial examining the effect of financial navigation on financial toxicity in adult patients with cancer in rural and non-rural settings
Source: Trials. 2022 Oct 3;23:839. doi: 10.1186/s13063-022-06745-4 (PMC9527389; doi:10.1186/s13063-022-06745-4)
Supplement: Supplementary file 7 — Additional file 7. Navigator Materials– includes a study schema designed to help navigators through the study process and a table of definitions given to navigators to ensure consistency and understanding across sites [file 13063_2022_6745_MOESM7_ESM.pdf]

Study Flow Diagram (from Standard Operating Procedures Manual for Financial Navigators)

The diagram below gives the overall study steps based on recruitment type (in person or by phone) depending on how participants prefer to complete forms. There are 4 ways patients can complete forms: on a tablet, by paper, by email, or you can read forms to the patient and directly enter their responses into REDCap.

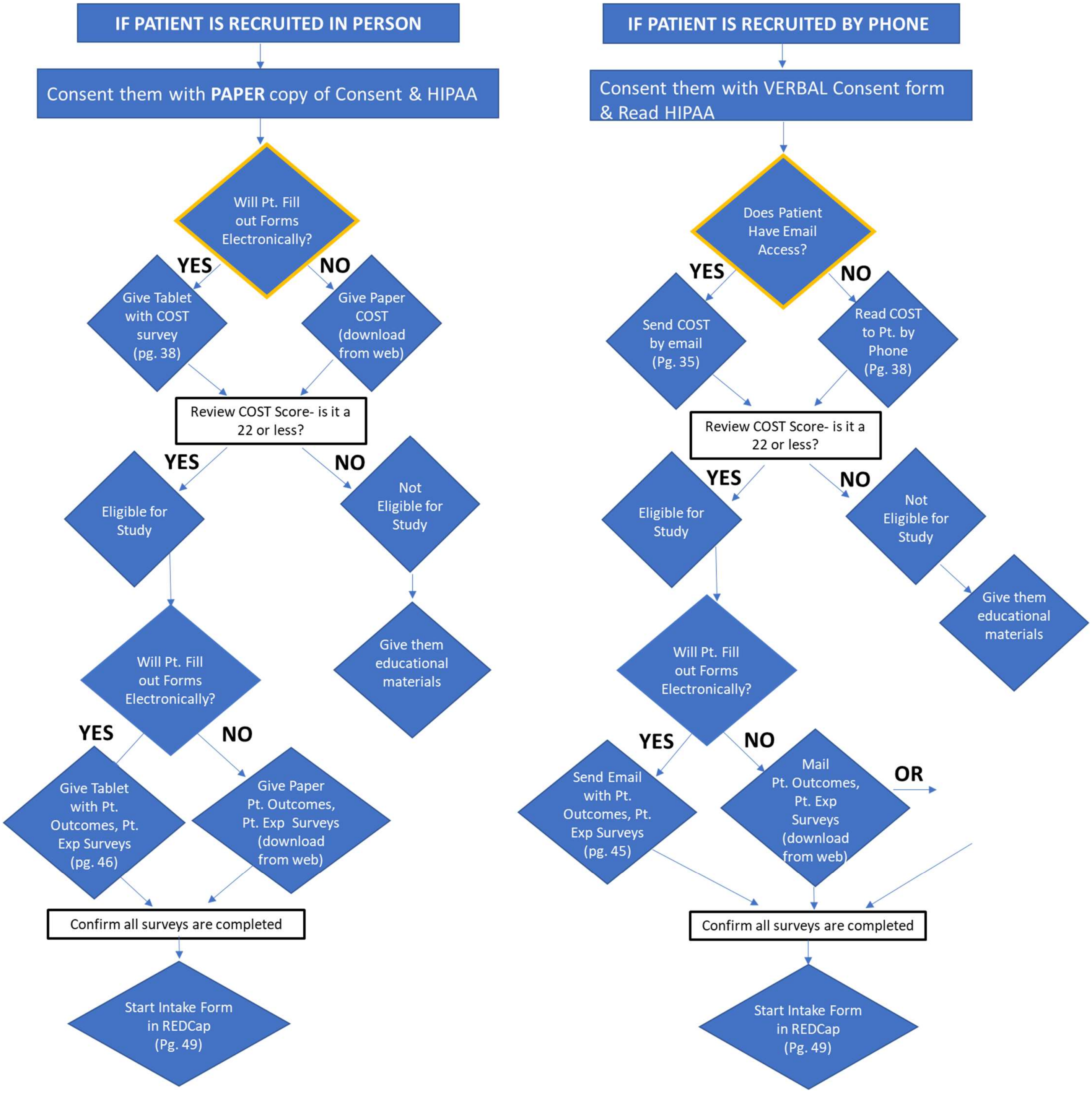

(Continued on next page)

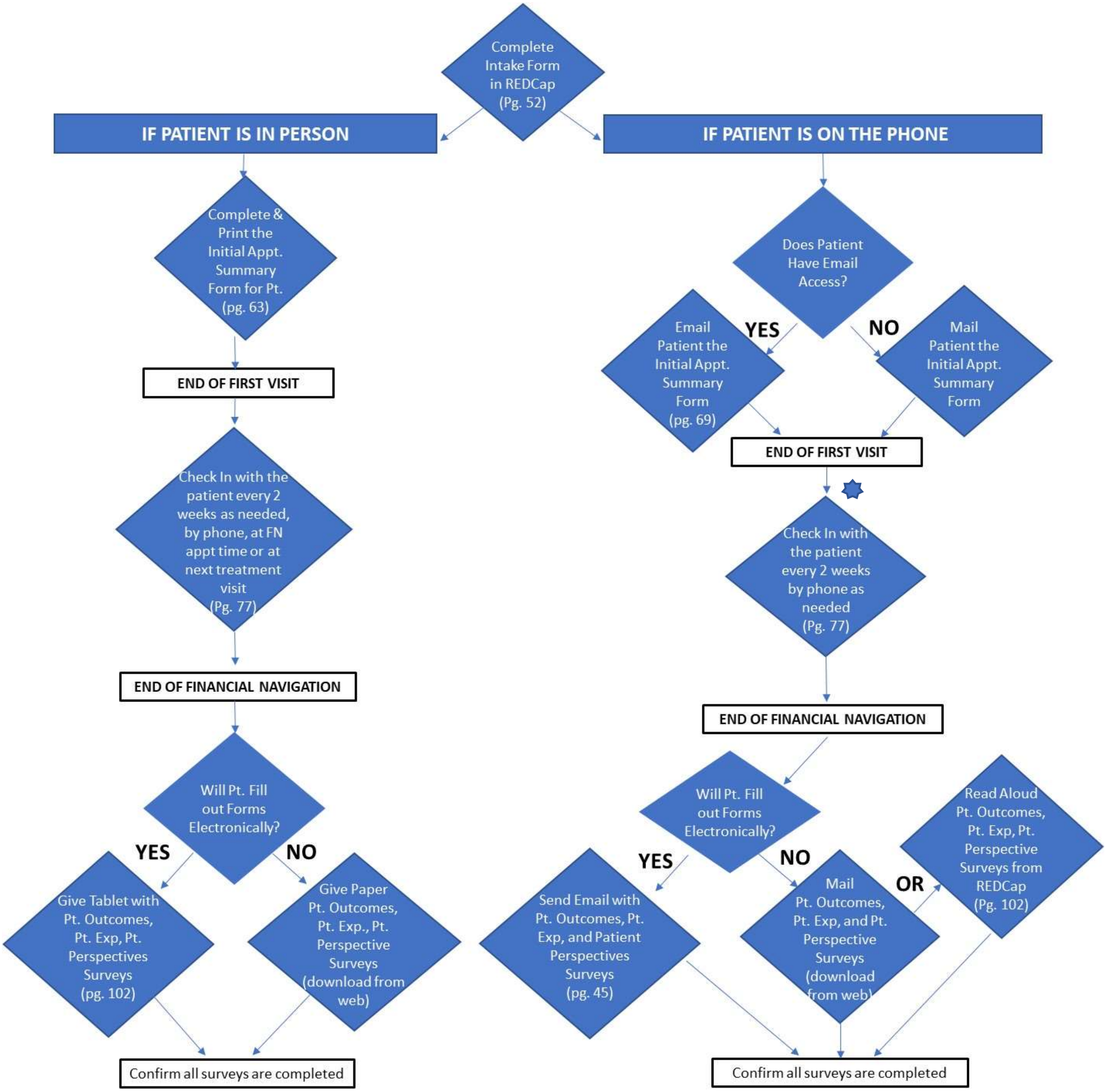

If you refer to PAF as part of your Financial Navigation process, they will also check in with the participant every 2 weeks. They will let you know when they are done navigating by completing the 'end of navigation' date in the PAF form.

STUDY DEFINITIONS

Term	Definition
Informed Consent	The act of obtaining someone's voluntary and informed agreement to participate in the study. Informed consent is essential for most clinical studies, including the LIFT Study.
Evidence-Based Intervention	A treatment or program based on sound scientific methods that has been proven to be effective.
Financial Navigation	Financial navigation (FN) is one type of evidence-based program implemented at the clinical practice or system level that identifies patients at high risk for financial toxicity (FT), assesses eligibility for existing federal, nonprofit, manufacturer, and local financial support resources, clarifies treatment cost expectations, and develops strategies to cope with high costs of care.
Financial Navigator	A person designated to support a patient and his/her loved ones through the financial assistance process over a sustained period of time.
Financial Toxicity	A term used to describe the high cost and cost-related psychosocial burden that cancer care places on patients and their families.
Eligibility Criteria: Inclusion Criteria	Inclusion criteria are defined as the key features of the cancer patient that are required for study participation. Typical inclusion criteria include age, language, medical history, and current symptoms or issues that may be targeted in the study.
Eligibility Criteria: Exclusion Criteria	In contrast, exclusion criteria are defined as features of the potential study participants who cannot participate in the study. See this study's eligibility criteria (Pg 14)
Institutional Review Board (IRB)	A group of people that reviews clinical study descriptions, or protocols, to ensure the protection of human subjects including but not limited to reviewing the risks and benefits of the study, the ethical treatment of patients, and protection against coercion to participate in the study.
Patient Outcomes	In this manual, this refers to the group of participant surveys that ask questions related to someone's quality of life, like physical health, emotional concerns, or transportation needs. These kinds of surveys are also referred to as 'patient outcomes' surveys.
Participant	Participant is another name for a study subject. In this case, participant refers to the patient with cancer (or survivor) who is participating in the study. For this manual, we will use both the word participant and patient depending on the reference point.
Principal Investigator (PI) and Co-PI	A **Principal Investigator** or PI is the individual responsible for the preparation, conduct, and administration of a research grant, cooperative agreement, training or public service program, contract, or other sponsored study.<br><br>**A Co-PI** has responsibilities similar to that of a PI on research studies. While the PI has ultimate responsibility for the conduct of a research study, the Co-PI/Co-I is also obligated to ensure that the study is conducted in compliance with applicable laws, regulations and institutional policy governing the conduct of research.

Protocol	A research protocol is a document that outlines the study plan. The plan must be designed to answer the research question and it must also provide a detailed description of the objectives, design, methodology, statistics, and protections employed. The protocol in effect is the cookbook for the study.
Protocol Review Committee (PRC)	The Lineberger Comprehensive Cancer Center (LCCC) has a protocol review committee, which is similar to an IRB but this committee reviews the scientific merit of the study and all study procedures whereas the IRB focuses more on the protection of study participants.
Standard Operating Procedure (SOP)	A standard operating procedure is a set of step-by-step instructions compiled by an organization to help workers carry out complex routine operations. SOPs aim to achieve efficiency, quality output and uniformity of performance, while reducing miscommunication and failure to comply with industry regulations.
